# Metabolic deficiencies underlie reduced plasmacytoid dendritic cell IFN-I production following viral infection

**DOI:** 10.1038/s41467-025-56603-5

**Published:** 2025-02-07

**Authors:** Trever T. Greene, Yeara Jo, Carolina Chiale, Monica Macal, Ziyan Fang, Fawziyah S. Khatri, Alicia L. Codrington, Katelynn R. Kazane, Elizabeth Akbulut, Shobha Swaminathan, Yu Fujita, Patricia Fitzgerald-Bocarsly, Thekla Cordes, Christian Metallo, David A. Scott, Elina I. Zúñiga

**Affiliations:** 1 Department of Biological Sciences, University of California, San Diego, La Jolla, CA USA; 2 Department of Pathology, Immunology and Laboratory Medicine, Rutgers New Jersey Medical School, Newark, NJ USA; 3 Department of Medicine, Division of Infectious Disease, The State University of New Jersey, Rutgers, New Jersey Medical School, Newark, NJ USA; 4 Division of Next-Generation Drug Development, Research Center for Medical Sciences, The Jikei University School of Medicine, Tokyo, Japan; 5 Department of Bioinformatics and Biochemistry, Braunschweig Integrated Centre of Systems Biology (BRICS), Technische Universität Braunschweig, Braunschweig, Germany; 6 Department of Bioengineering, University of California, San Diego, La Jolla, CA USA; 7 Molecular and Cell Biology Laboratory, Salk Institute for Biological Sciences, La Jolla, CA USA; 8 Cancer Center, Sanford Burnham Prebys Medical Discovery Institute, La Jolla, CA USA

**Keywords:** Plasmacytoid dendritic cells, Infection, Interferons, Nutrient signalling

## Abstract

Type I Interferons (IFN-I) are central to host protection against viral infections, with plasmacytoid dendritic cells (pDC) being the most significant source, yet pDCs lose their IFN-I production capacity following an initial burst of IFN-I, resulting in susceptibility to secondary infections. The underlying mechanisms of these dynamics are not well understood. Here we find that viral infection reduces the capacity of pDCs to engage both oxidative and glycolytic metabolism. Mechanistically, we identify lactate dehydrogenase B (LDHB) as a positive regulator of pDC IFN-I production in mice and humans; meanwhile, LDHB deficiency is associated with suppressed IFN-I production, pDC metabolic capacity, and viral control following infection. In addition, preservation of LDHB expression is sufficient to partially retain the function of otherwise exhausted pDCs, both in vitro and in vivo. Furthermore, restoring LDHB in vivo in pDCs from infected mice increases IFNAR-dependent, infection-associated pathology. Our work thus identifies a mechanism for balancing immunity and pathology during viral infections, while also providing insight into the highly preserved infection-driven pDC inhibition.

## Abstract (web summary)

Plasmacytoid dendritic cells (pDC) are the major IFN-I-producing cells, but this production returns to baseline soon after viral infection. Here the authors show that this decrease in IFN-I production and related pDC functions may be attributed to suppressed oxidative and glycolytic metabolism of pDCs, with lactate dehydrogenase B identified as a regulator.

## Introduction

Persistent infections, including human immunodeficiency virus (HIV), hepatitis C virus (HCV), hepatitis B virus (HBV), *Mycobacterium tuberculosis*, and malaria represent massive burdens on human health. A key hallmark of these infections is immunosuppression, which limits responses against these pathogens, but also against unrelated secondary infections, cancer, and vaccination^1–3^. While immune suppression in this context can be beneficial to the pathogen, it also represents an adaptation by the host immune system that limits immunopathology and enables long-term host survival. Indeed, ablation of key immunosuppressive molecules often results in host death during infection even as control of the pathogen is improved^1,2^. Short-term alleviation of immunosuppressive adaptations has also shown therapeutic promise, allowing for clearance of pathogens and improved control of cancer^1,4–6^. These adaptations have been well studied in T cells and B cells where persistent stimulation promotes functional exhaustion^4,7^. However, relatively little is understood about the mechanisms that drive adaptations within the innate immune system.

Type I interferons (IFN-Is) are a family of key anti-viral and anti-neoplastic cytokines that act via limiting spread of viruses, restricting replication of transformed cells, as well as by enhancing innate and adaptive responses against viruses and tumors^8–10^. As specialized IFN-I producing cells, plasmacytoid dendritic cells (pDCs) rapidly respond to viral infection by synthesizing and releasing large quantities of IFN-I^11–13^. Through this function pDCs are known to promote the control of many viruses^11–13^. For instance, genetic deficiencies in virus sensing and IFN-I production by pDCs are associated with severe COVID-19 (reviewed in ref. ^14^). However, after an initial robust response, pDCs lose their capacity to produce interferon in response to the ongoing infection or secondary stimulation (reviewed in refs. ^15–17^). Impaired IFN-I production, which is observed a few days after both acute and persistent viral infection and is sustained in the chronic setting, associates with compromised responses against unrelated secondary infections in both mice^18,19^ and humans^16,17^. Similar phenotypes have been described in several human and mouse cancer models as well^20–23^ and promoting pDC IFN-I production capacity has been proposed as a means of overcoming cancer immunosuppression^24–27^. However, little is understood about the molecular processes that initiate and sustain this loss of pDC IFN-I production capacity of this highly conserved phenomenon is entirely unknown.

There are several mechanisms by which pDC-derived IFN-I can be suppressed following infection (reviewed in refs. ^12,14,15,28^). One such mechanism is immune contraction, that may occur after a wave of immune activation, that leads to reduced number of immune cells, including pDCs^14,15,28^. Another distinct process is immunosuppression, an active strategy aimed at generating an inhibitory environment that dampens the immune response, as it is, for example the repression of pDC-IFN-I production mediated by inhibitory cytokines^14,15,28^. Additionally, numerous viruses encode proteins that can directly suppress IFN-I production in the host’s cells, which often requires productive cell intrinsic viral replication^14,29^. Lastly, “exhaustion” refers to the decline in immune functionality after an initially strong and/or sustained cell-intrinsic stimulation through pathogen recognition receptors (e.g., TCR, TLR)^7,30–32^.

In our previous work, we demonstrated that following lymphocytic choriomeningitis virus (LCMV) infection, pDC IFN-I suppression does not rely on a number of cell-extrinsic factors that may be immunosuppressive such as T cells, IFNγ, IFN-I, TNFα, or IL-10^19^. Instead, the pDC loss of function is partly dependent on cell-intrinsic TLR7 stimulation, and dissociated from the pDC-intrinsic viral loads or the infectious environment, which led us to coin the term pDC exhaustion^28,30^.

In the present study we improve our understanding of the balancing forces involved in regulating pDC derived IFN-I throughout viral infection. Characterizing the transcriptional landscape of pDCs throughout LCMV infection identifies a’pDC exhaustion signature‘ and uncovers metabolic pathway alterations that associate with pDCs’ reduced IFN-I production capacity. We reveal significant reductions in metabolism in pDCs, show that the metabolic enzyme lactate dehydrogenase B (LDHB) is downregulated in mouse and human pDCs exhibiting infection-driven loss of function, and find that LDHB supports IFN-I production in pDCs in vitro and in vivo. As such, chimeric mice with LDHB deficiency in the pDC compartment exhibited compromised viral control after coronavirus infection, demonstrating the importance of LDHB in pDCs for optimal antiviral responses, and that preserving LDHB expression and pDC function in infected mice associates with IFNAR-dependent infection-induced pathology in the colon. Altogether, our results identify a key role for LDHB in balancing pDC antiviral function and immunopathogenic potential during infection.

## Results

### Transcriptional analysis of pDCs from virally infected mice identifies multiple pathways associated with their IFN-I exhaustion

We and others have previously described in vivo pDC loss of function by using LCMV infection in its natural murine host as a model system^18,19,30^. While both acute and chronic infections with LCMV cause pDC suppression after a few days, only the persistent LCMV variant, Clone 13 (Cl13) sustains this phenotype for up to at least 30 days post infection (p.i.)^18,19,30^. We have previously established that this is highly dependent on cell intrinsic signaling through TLR7^30^ and independent of many cell-extrinsic factors (IFNγ, IFN-I, TNFα, and IL-10)^19^. However, we had not previously shown whether this depends on glucocorticoids, which are known to be elevated after LCMV infection^33^. To test this, we made use of adrenalectomized (ADX) mice, which lack the ability to produce glucocorticoids. Briefly, ADX or sham operated (SHAM) mice were infected with LCMV Cl13, their splenocytes isolated, and stimulated with CpG, and cytokine production in pDCs measured by flow cytometry. Because ADX mice die early after LCMV infection^34^ these experiments were performed at day 4 p.i. Notably pDCs were equally suppressed in their IFNα production capacity in the spleens of ADX and SHAM mice suggesting glucocorticoids do not play a major role in the establishment of pDC inhibition after infection (Supplementary Fig. 1).

**Fig. 1:**
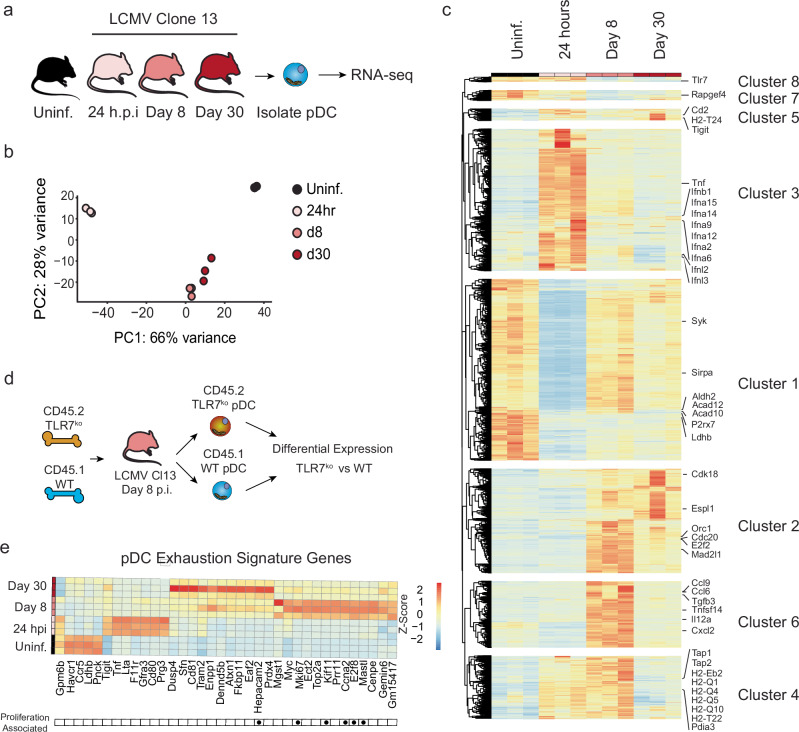
Defining the Transcriptome changes of pDCs throughout LCMV Infection. **a** Experimental design for pDC RNA seq. pDC (Lineage^−^, CD11c^+^, B220^+^, BST2^+^) were isolated from Uninfected or LCMV Cl13 infected mice at day 1 (24 h), 8, or day 30 p.i. and subjected to RNA-seq. **b**. Principal component (PC) plot showing pDC from uninfected mice, or from mice at day 1 (24 h), 8, or 30 p.i. **c** Hierarchical clustering of all genes that were DE between pDC from uninfected mice or from mice at day 1 (24 h), 8, or 30 p.i. **d** Experimental design for analysis of TLR7 dependent gene expression analysis. 50:50 TLR7^−/−^:WT mixed bone marrow chimeras were generated and infected with LCMV Cl13. At day 8 p.i., WT (Lineage^−^, CD11c^+^, B220^+^, BST2^+^, CD45.1^+^) or TLR7^−/−^ (Lineage^−^, CD11c^+^, B220^+^, BST2^+^, CD45.2^+^) were isolated and subjected to Microarray analysis. **e** Expression of genes differentially regulated in both TLR7^−/−^ vs WT pDC (from d) and in day 8 & 30-infected vs. uninfected mice (from a). Gene expression before and at different times after infection is depicted. Genes associated with cell cycle (GO: 0007049) are indicated by black dots.

LCMV Cl13 establishes a systemic infection, with the virus detectable in most tissues, including the bone marrow, blood and spleen, within 1–2 days p.i., and persisting for 2–3 months at high titers^35–38^. Consistent with this, our previous work shows that pDCs in the spleen, bone marrow, peripheral, and mesenteric lymph nodes upregulate IFN-I as early as day 1 p.i^39^, before they become suppressed by day 5 and up to at least 30 p.i^19,30^. Thus, to characterize the transcriptional changes that take place in pDCs during infection, and highlight pathways associated with pDC peak IFN-I production (i.e., 24 h p.i.) and subsequent suppression, we isolated pDCs from the spleens of uninfected mice or mice at days 1, 8 and 30 after LCMV Cl13 infection (Fig. 1a) and subjected them to RNA-seq. We observed many differentially expressed genes (DEGs) between uninfected and each timepoint p.i. (Adjusted p-value of less than 0.05, Log2 Fold Change greater than 1), with the largest number of DEG observed at 24 h p.i. (4248, Supplementary Data 1), fewer DEG at day 8 p.i. (2492, Supplementary Data 2), and the least number of DEG at day 30 p.i. (1787, Supplementary Data 3). We observed a high level of overlap in DEG identified for each timepoint with respect to pDCs from uninfected mice ranging from 40% (when comparing day 1 and day 30) to 76% (when comparing day 8 to day 30) (Supplementary Fig. 2a). In line with this observation, principal component analysis (PCA) showed that, while each of these conditions clustered separately, day 8 and day 30 p.i. were more similar to each other than they were to day 1 p.i. (Fig. 1b). This likely represents differences between IFN-I producing pDCs (day 1 p.i.)^39^ and suppressed pDCs (day 8 and day 30 p.i.)^18,19,30^.

Gene set enrichment analysis (GSEA) for DEG at 24 h when compared to those from uninfected animals revealed 60 pathways significantly enriched (adj. *p* < 0.05) (Supplementary Table 1). These were largely associated with various viral and inflammatory diseases, pattern recognition, and cytokine production or signaling (e.g., Human papillomavirus infection, Human cytomegalovirus infection, Coronavirus disease, Epstein-Barr virus infection, HIV-1 infection, KSHV infection, Influenza A, Hepatitis B, Hepatitis C, Measles, Toll-like receptor signaling pathway, Cytokine-cytokine receptor interaction, RIG-I-like receptor signaling pathway) and are likely representative of the anti-viral response these pDCs are actively engaged with at this timepoint^19,39^. As expected, given their lower number of DEG when compared to pDCs from uninfected mice, pDCs from day 8 and day 30 p.i. showed fewer significantly enriched pathways (18 and 10 respectively) (Supplementary Tables 2–3) versus pDCs at day 1 p.i. Day 8 p.i. pathways included several related to intracellular transport (e.g., regulation of actin cytoskeleton, axon guidance, synaptic vesicle cycle, nucleocytoplasmic transport) suggesting there may be changes in vesicle transport or the arrangement of the actin cytoskeleton of pDCs at this timepoint, and consistent with previously described morphological changes that pDCs undergo following stimulation^40^. We also observed several diverse metabolism related pathways (Fatty acid biosynthesis, Choline Metabolism in cancer, Cholesterol metabolism, Terpenoid backbone biosynthesis, glycosaminoglycan degradation) suggesting the metabolism of these cells may be altered. On the other hand, at day 30 p.i. we observed changes in metabolic pathways such as Vitamin B6 metabolism, Tyrosine metabolism, Linoleic acid metabolism, Taurine and hypotaurine metabolism, along with several disease related pathways that may represent the persistence of inflammation in this model (Legionellosis, Amyotrophic lateral sclerosis).

One strength of this data set is that it allows us to explore how pDC gene expression may change over time throughout infection. Therefore, to determine how changes in gene expression may regulate pDC function throughout infection we investigated the total set of DEG between the uninfected condition and all 3 infection timepoints (5361 unique genes). We then subjected these genes to hierarchical clustering analysis to identify temporally associated clusters of gene expression throughout infection (Fig. 1c). We then separated this into 8 clusters with unique temporal characteristics based on analysis of the within-cluster sum of squares^41,42^ (Supplementary Fig. 2b). For example, cluster 3 identified genes that were transiently increased at 24 h p.i. but returned to normal levels at day 8 and day 30. This includes most *Ifna* isoforms, *Ifnl2*, *Ifnl3*, *Ifnb1*, *Tnf*, and numerous other chemokines and cytokines (Supplementary Data 4) representing the acute pDC anti-viral response that is absent in suppressed pDCs at later stages of infection. Cluster 1 which represents genes that are mostly reduced through the entire course of infection (Supplementary Fig. 2b) includes many metabolism related genes (e.g., Aldh2, Acad12, Acad10, P2rx7, Ldhb and others, Supplementary Data 4). Cluster 2 which represents genes increased at days 8 and 30 p.i. is highly enriched for genes associated with cell-replication (e.g., Orc, Cdk18, Cdc20), in line with our previous observations that pDCs at day 8 and day 30 gain proliferative potential^30^. Cluster 4, which is made up of genes that are increased in expression at 24 h p.i. and 8 days p.i., but not at day 30 p.i. includes many antigen presentation associated proteins (Tap1, Tap2, H2-Eb2, H2-T22), in line with previous observations that pDCs gain some antigen presenting potential following initial stimulation^12^. Finally, cluster 6 includes genes specifically high at day 8, but not other timepoints. This notably contains a set of cytokines and chemokines which are not highly upregulated during the acute response at 24 h p.i., but are at day 8 p.i. (e.g., Tgfb3, Il12a, Ccl6, Ccl9, Cxcl2).

We then sought to identify genes and pathways that associate specifically with pDC suppression. For this we investigated the overlap between DEG at day 8 p.i. and day 30 p.i. as compared to pDCs from uninfected mice. We subjected this gene list to overrepresentation analysis of gene ontology (GO) terms. To restrict to the most significant pathways we considered only pathways with 5 or more genes identified, and an adjusted p-value less than 0.1. This identified 1260 pathways (Supplementary Data 5), though notably these were largely overlapping in scope with many, as expected based on previous work, being related to cellular proliferation, and anti-pathogen responses. To refine this analysis, we then made use of simplifyEnrichment^43^ to perform semantic similarity analysis and identify highly similar pathways. Using the binary cut method^43^ to determine clusters, this analysis identified 16 clusters of GO pathways (Supplementary Data 5) which share semantic similarity. Of these, 13 were large enough to be associated with common keywords shared by those pathways (Supplementary Fig. 2c), while the remainder included too few pathways for this type of analysis. As expected, several clusters were enriched for terms relating to the cell cycle and survival (e.g., chromosome, segregation, cycle, mitotic, checkpoint, proliferation, apoptosis), as well as a cluster enriched for terms relating to immune responses (immune, lymphocyte, interleukin). There were also several clusters related to cell-cell interaction (adhesion, cell-cell, junction, integrin), potentially relating to the role of pDCs in forming interferogenic-synapses during infection^44–52^. Finally, we also found a collection of pathways relating to cellular metabolism (metabolic), again reinforcing the potential for changes in metabolism in suppressed pDCs. Altogether these data bolster previous observations that pDCs gain proliferative capacity^30^, and increase apoptosis^53^ following infection. Furthermore, these data suggest that pDC loss of function after infection may be associated with alterations in their cell-cell interactive properties and/or metabolic capacities which have not yet been described.

We then sought to further refine our analysis to specifically identify genes associated to cell-intrinsic-TLR7-mediated pDC exhaustion^30^. To this end, we analyzed the differences in pDC gene expression in TLR7 knockout (TLR7^−/−^) and wild type (WT) pDCs from LCMV Cl13 infected mixed bone marrow (BM) chimeras (Fig. 1d), in which both WT and TLR7^−/−^ pDCs are exposed to the same infectious environment^30^. In this case the WT pDCs are suppressed, while the TLR7^−/−^ pDCs retain their function^30^. Direct analysis between these groups identified 110 DE genes, (Adjusted *p* < 0.1, Log_2_ (Fold Change)) with magnitude more than 0.75, Supplementary Data 6. We then compared these genes to the set of genes that are persistently differentially expressed between pDCs from uninfected animals (functional pDCs) and those from day 8 or day 30 (suppressed pDCs). Altogether this identified 36 genes that met these criteria. This “pDC exhaustion signature”, includes several notable genes that have previously been observed to be altered in suppressed pDCs during SIV infection (*Tigit, Ccr5*)^54^, as well as molecules known to regulate pDC function (*Cd81*^55,56^, *Myc*^57^) (Fig. 1e). Additionally, this signature contains several cell-cycle related genes (GO: 0007049, Fig. 1e), in line with our previous observation that acquired proliferative capacity in pDCs from infected mice is partially TLR7 dependent^30^. All together these data provide a resource of gene expression data for pDC peak IFN-I response and subsequent pDC suppression during a natural viral infection, including the first defined pDC exhaustion signature, which may be useful for the identification of pDC regulators in future studies.

### LCMV infection drives a persistent reduction in metabolic capacity of pDCs

**Fig. 2:**
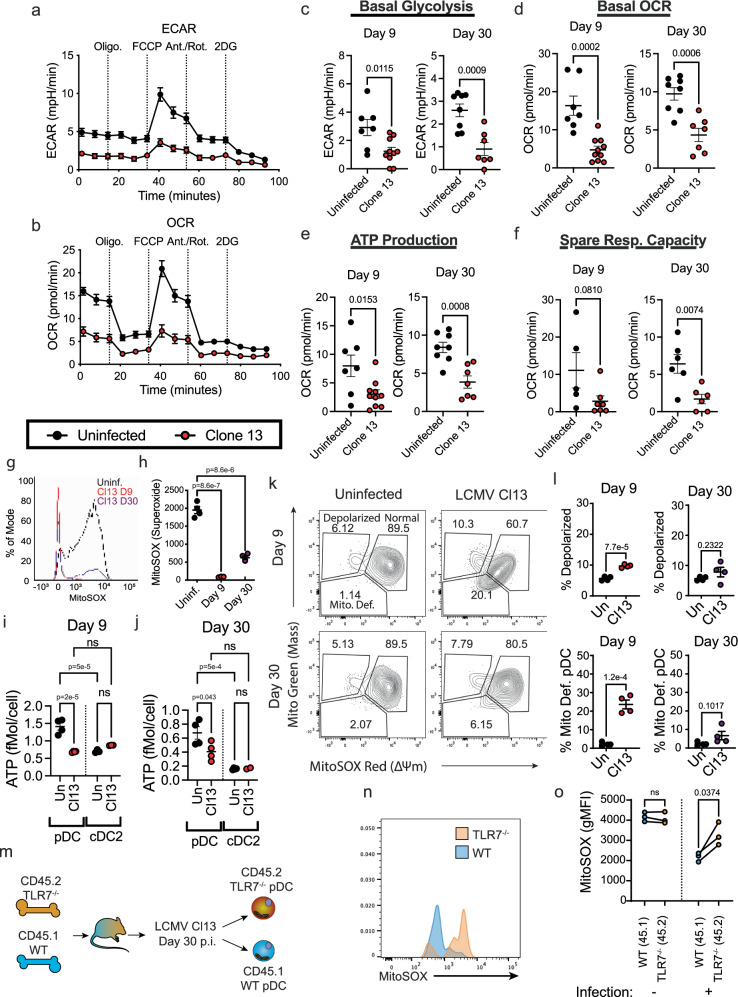
Clone 13 infection drives long-term changes in metabolism of pDCs and short-term changes in mitochondrial content. Seahorse assay traces of extracellular acidification rate (ECAR) (**a**) and oxygen consumption rate (OCR) (**b**) of pDCs isolated from uninfected (black) or LCMV Cl13 infected (red) mice at day 30 p.i. Derivative measures of ECAR and OCR from Seahorse assay at day 9 and day 30 p.i. (red) compared to uninfected mice (black) **c** Basal Glycolysis **d** Basal OCR **e** ATP production, **f** Spare Respiratory Capacity **g** representative stain of MitoSOX in pDC from uninfected mice (black) and LCMV Cl13 infected mice at day 9 (red) or day 30 p.i. (purple) (**h**) Quantification of MitoSOX gMFI from pDCs. Total per-cell ATP content in pDC and cDC2 from uninfected mice or LCMV Cl13 infected mice at day 9 (**i**) or day 30 p.i. (**j**). **k** Representative staining of mitochondrial mass (MitoGreen) and charge (MitoSox) from pDCs at indicated days p.i. **l** Quantification of percentage of pDCs with depolarized mitochondria (top) or that are mitochondria deficient (bottom) at indicated days p.i. **m** Experimental outline for panels n,o. 50:50 TLR7^−/−^:WT mixed bone marrow chimeras were generated and infected with LCMV Cl13. At day 30 p.i., WT (Lineage^−^, CD11c^+^, B220^+^, BST2^+^, CD45.1^+^) or TLR7^−/−^ (Lineage^−^, CD11c^+^, B220^+^, BST2^+^, CD45.2^+^) pDCs were analyzed for MitoSox staining via FACS. **n** Representative staining of MitoSOX in TLR7^−/−^ and WT pDCs from (**m**), **o** Quantification of MitoSOX levels in WT & TLR7^−/−^ pDCs from m. Data are pooled from 3 (**a**, **b**), 4 (**c**, **d**, **e**, **f**), or representative of 2 (**g**, **h**, **k**, **o**) or 3 (**i**, **j**) independent experiments. Data are shown as mean ± SEM. Statistics used are Two tailed Student’s *t*-test **(c**–**f**, **l)**, One way ANOVA with Tukey Correction (**h**), or Two tailed Paired *t*-test (**o**).

It has previously been appreciated that metabolism regulates pDC capacity to produce IFN-I^58–60^, however no study has so far investigated the long-term impact of viral infection on pDC metabolism. Given our analysis identified metabolism as a recurrent theme in pathways changed in pDCs exhibiting functional loss (Supplementary Tables 2, 3, Supplementary Fig. 2c), we sought to determine if the metabolism of suppressed pDCs was significantly altered during infection. For this we used the Agilent Seahorse platform to evaluate oxygen consumption rate (OCR) as a proxy for OxPhos, and extracellular acidification rate (ECAR) as a proxy for lactate terminal glycolysis^61^. C57BL/6 mice were infected with LCMV Cl13 or left uninfected. On day 9 or 30 (p.i.), we isolated pDCs from the BM of these mice and evaluated their basal ECAR and OCR. We observed a significant reduction in both basal ECAR (Fig. 2a, c) and basal OCR (Fig. 2b, d) in pDCs from infected mice in both the acute (day 9 p.i.) and chronic (day 30 p.i.) phases of LCMV Cl13 infection. This was coordinate with a reduction in ATP production defined as the amount of OCR lost after ablating ATP synthase activity with Oligomycin (Fig. 2e). Spare respiratory capacity (SRC) or the difference between basal OCR and the maximal OCR measured by decoupling mitochondrial electron transport with FCCP (Fig. 2f) was also reduced. This suggests both base-line OxPhos activity and pDC potential for oxygen consumption are severely restricted in suppressed pDCs compared to their functional counterparts. Intriguingly, we did not observe any differences in OCR in the first 3 h following stimulation of either functional or suppressed pDCs with CpG-A (Supplementary Fig. 3a), this differs from what has been observed in humans which have showed an increase in OCR following stimulation with either HSV or Influenza Virus from a starting point of 4 h after stimulation^60^. As we measured changes in OCR from initiation of stimulation, the difference may represent differences in timing of measurement. Alternatively, this could also be a functional difference between mouse and human pDCs, or a difference in response to diverse stimuli. As a parallel measure of mitochondrial activity we assessed levels of mitochondrial associated superoxide (mtSOX) in pDCs using a superoxide specific mitochondrially associated dye (MitoSOX Red), and found that mtSOX was much lower in pDCs from LCMV Cl13 infected mice at both day 9 and day 30 p.i. (Fig. 2g, h). Thus, our results suggest that the capacity to engage OxPhos as well as glycolysis is compromised in suppressed pDCs.

We then sought to determine whether ATP levels were altered in pDCs from infected mice. For this we used a luminescence-based assay of ATP content, comparing pDCs from uninfected mice with pDCs from infected mice at day 9 or day 30 p.i. (Fig. 2i, j). We observed a striking decrease in total ATP content in pDCs at both days in infection, supporting our Seahorse assay observations and further demonstrating that suppressed pDC are metabolically unfit. Importantly, this is not a general phenomenon as conventional (c)DC from these same mice did not show any appreciable difference in ATP content (Fig. 2i, j). Notably, we also observed that, on a per-cell basis functional pDCs from uninfected mice, but not suppressed, pDCs showed higher levels of ATP than cDCs (Fig. 2i, j).

To determine if the changes in metabolism we observed in pDCs during chronic viral infection were the result of changes in mitochondrial content or function we assessed mitochondrial content (mtMass) and mitochondrial charge (mt∆ψ) by flow cytometry. Mitochondrial mass and charge were evaluated together using charge dependent (MitoTracker Red chloromethyl-X-rosamine (CMXRos)) and independent (MitoTracker Green FM) dyes. In uninfected animals the majority of pDCs had similar mtMass and mt∆ψ, with a small population showing high mass and low mt∆ψ likely representing pDCs with depolarized mitochondria (Fig. 2k, top-left), as has previously been described in exhausted T cells^62^. This population expanded significantly in the early stages (day 9 p.i.) of LCMV Cl13 infection (Fig. 2k, l), but was not significantly different at day 30 p.i (Fig. 2k, l), although average levels were still slightly higher than baseline at this later time point. Additionally, during LCMV Cl13 infection a third population emerged, showing low mtMass and mt∆ψ as compared to pDCs from uninfected mice (Fig. 2k, top-right). As this population retained proportionality between mtMass and mt∆ψ it is unlikely these are depolarized, but instead have reduced mitochondrial mass (Fig. 2k, l). We refer to these cells as mitochondria deficient pDCs. By day 30 p.i. the number of mitochondria deficient pDCs were again low, although this remained slightly but not significantly higher than baseline (Fig. 2l). Therefore, changes in mitochondrial content may at least partially explain the loss of OxPhos observed at day 9 after LCMV Cl13 infection but are unlikely to fully explain the sustained loss of OxPhos that associates with long-term pDC suppression observed at day 30 p.i.

While these data correlate loss of OCR and ECAR in mitochondria to the timeline of pDC loss of function, we sought to determine if the reduction in mitochondrial activity observed at day 9 at 30 p.i. was also dependent on TLR7 and therefore may relate to pDC exhaustion. For this we again generated TLR7^−/−^:WT mixed BM chimeras, and analyzed the level of mtSOX in each compartment at day 30 p.i. (Fig. 2m). We observed no differences in mtSOX levels in WT and TLR7^−/−^ pDCs prior to infection, but mice infected with LCMV Cl13 showed reduced levels of mtSOX specifically in the WT compartment (Fig. 2n, o) suggesting that, like functional exhaustion^30^, loss of mtSOX production is cell intrinsically dependent on TLR7 signaling in pDCs.

### LDHB in pDCs promotes IFN-I production and viral control

**Fig. 3:**
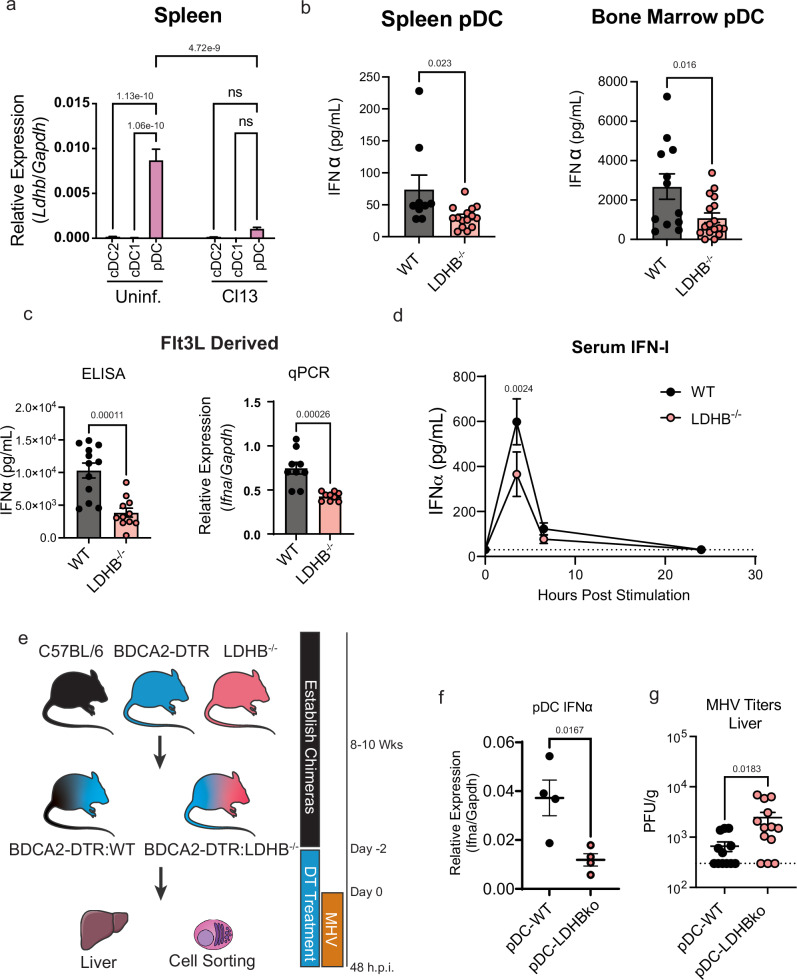
LDHB is essential for optimal mouse pDC IFN-I production in vitro and in vivo. **a** Relative expression of LDHB was evaluated by RT-qPCR in purified cDC2, cDC1, and pDC from the spleens of uninfected and LCMV Cl13 infected mice at day 9 p.i. **b** ELISA quantification of IFNα secreted by pDC isolated from the spleen (left) or bone marrow (right) of WT or LDHB^−/−^ mice after 12 h stimulation with CpG-A. **c** ELISA (left) or qPCR (right) quantification of secreted IFNα or *Ifna* transcript in pDC isolated from Flt3L cultures derived from WT or LDHB^−/−^ mice after 12 h stimulation with CpG-A. **d** ELISA quantification of IFNα in the serum of WT (black) or LDHB^−/−^ (red) mice treated with CpG-A. **b**–**d** Unstimulated pDC were below the limit of detection. **e** Experimental design for f-g, 50:50 mixed bone marrow chimeras for BDCA2-DTR:WT or BDCA2-DTR:LDHB were generated, then treated with DT daily from two days prior to infection to deplete BDCA2 expressing pDCs. Mice were then infected with MHV, 48 h.p.i pDCs were sorted and virus titers were quantified. **f** Expression of *Ifna* transcript in pDC from e. **g** Plaque assay quantification of MHV in livers from e. Data are pooled from 2 (**d**), 3 (**c** right, **g**), 4 (**c**, left), or 5 (**b**) independent experiments or representative of 2 (**a**) or 3 (**f**) independent experiments. Data are shown as mean ± SEM. Statistics used are One way ANOVA with Tukey Correction (**a**), Two tailed Student’s *t*-test (**b**, **c**, **f**, **g**)¸ multiple unpaired two tailed *t*-tests with two stage step up FDR (**d**).

Given the reductions in pDC OxPhos and glycolysis during sustained infection we sought to investigate genes within the aforementioned “pDC exhaustion signature” (Fig. 1e) that have established roles in glycolysis as well as the TCA cycle and/or electron transport (Reactome R-MMU-1428517.1). Among the genes in the pDC exhaustion signature (Fig. 1e) only Lactate dehydrogenase B (*Ldhb*) met these criteria. Additionally, *Ldhb* is specifically highly expressed in pDCs as compared to cDCs in uninfected mice (Fig. 3a), and it is strongly and persistently downregulated during LCMV infection (Fig. 3a, Supplementary Fig. 3b) suggesting it may represent a reasonable candidate regulator of pDC function. LDH enzymes composed of LDHB show slower V_max_ for pyruvate reduction to lactate than those comprised primarily of LDHA, and a strong substrate inhibition by pyruvate^63,64^. As such LDHB is primarily expressed in aerobic tissues where it is thought to promote OxPhos via the conversion of lactate to pyruvate^63^, although it is important to also acknowledge that under specific conditions LDHB also supports lactate terminal glycolysis^65,66^. Notably, we observed no significant change in the expression of *Ldha* in pDCs throughout the course of infection (Supplementary Data 1–3).

Given that there is no relationship defined for LDHB with respect to pDC function we sought to first determine if LDHB regulated pDC IFN-I production. For this we used LDHB knockout mice (LDHB^−/−^) generated by the International Mouse Phenotyping Consortium^67^. We first characterized the DC compartments of these mice and found no differences in total pDC or cDC numbers or their MHC-II expression, indicating that LDHB deficiency does not alter the number or maturation of DCs at steady state in the spleen (Supplementary Fig. 3c, d) or BM (Supplementary Fig. 3e, f).

To then investigate whether DC function was impacted by LDHB deficiency we FACS-purified pDCs or cDC2s from the spleens of LDHB^−/−^ animals or age matched WT controls, stimulated them with CpG-A and measured IFNα by ELISA. Under these conditions LDHB^−/−^ pDCs from the spleen and BM produced less IFNα than their WT counterparts (Fig. 3b), despite no changes in cell viability (Supplementary Fig. 3g). We observed similar results when pDCs were derived from day 8-Flt3L-cultures of LDHB^−/−^ BM (Fig. 3c), demonstrating that the aforementioned IFN-I reduction was not due to a potentially different environment in LDHB^−/−^ mice. Importantly, cDCs isolated from the same animals or from Flt3L-cultures showed no change in their IFNα production capacity (Supplementary Fig. 3h, i), demonstrating a pDC specific requirement for LDHB in IFN-I production. Additionally, quantification of *Tnfa* transcript in Flt3L derived pDCs showed no reduction in production of this cytokine (Supplementary Fig. 3j). This suggests there may be differences between LDHB deficiency and the state of pDC loss of function after infection, as suppressed pDCs can also show a defect in their capacity to produce TNFα^30^.

We then moved to determine if this observation could be extended in vivo. We made use of the synthetic TLR9 ligand CpG. IFN-I responses to CpG in mice peak quickly at 3.5 h and are pDC dependent^68,69^. WT or LDHB^−/−^ mice were injected with CpG, and IFN-I levels monitored in the serum. Despite similar number of pDCs in spleens and BM of WT and LDHB^−/−^ mice (Supplementary Fig. 3c, e), we observed significantly lower quantities of IFN-I in the serum of LDHB^−/−^ mice at the previously established peak of IFN-I production (3.5 h) (Fig. 3d) indicating that LDHB is necessary for optimal pDC IFN-I production in vivo.

To determine if LDHB in pDCs contributed to their capacity for IFN-I production during infection, and promoted anti-viral responses, we made mixed BM chimeras using a 50:50 ratio of BDCA2-DTR transgenic and LDHB^−/−^ or WT BM respectively (Fig. 3e). BDCA2-DTR mice express the diphtheria toxin (DT) receptor under the control of the human BDCA2 promoter and allow for the specific deletion of pDCs upon application of DT^70^. Thus, in these chimeras much of the immune compartment is composed of WT cells with normal function^70^, however, after DT treatment only pDCs from the non-DTR fraction (WT or LDHB^−/−^) remain (Supplementary Fig. 4a). Thus, we refer to these animals as pDC-WT and pDC-LDHB^−/−^.

We chose mouse hepatitis virus (MHV) as a model system to investigate the importance of LDHB in pDCs during an in vivo infection. MHV is a murine specific beta coronavirus and relative of human coronaviruses which cause severe acute respiratory syndrome (SARS-CoV-1, SARS-CoV-2) and middle east respiratory syndrome (MERS). As with SARS-CoV-1^71^, SARS-CoV-2^28^, and MERS^72^, MHV containment is highly dependent on pDC derived IFN-I^73^, and therefore a reasonable model virus to test the in vivo relevance of LDHB expression in pDCs.

At day 2 p.i., following depletion with DT we analyzed interferon expression in pDCs, and viral titers in the liver (Fig. 3f, g). In line with our in vitro and in vivo results with CpG stimulation (Fig. 3b–d) IFN-I production was significantly reduced in pDCs obtained from the pDC-LDHB^−/−^ versus pDC-WT infected mice (Fig. 3f). In conjunction with this we found reduced viral control (greater viral titers) in the livers of pDC-LDHB^−/−^ versus pDC-WT MHV infected mice (Fig. 3g), demonstrating that LDHB in pDCs promoted early MHV control. At this timepoint, we did not observe detectable viral titers in other organs for most mice (Supplementary Fig. 4b, c) although we did observe virus in the spleens and lungs of some, but not all pDC-LDHB^−/−^ mice (Supplementary Fig. 4b, c), indicating that while loss of control is not as complete as that observed with total IFNAR deficiency^73^ there may be a tendency toward broader organ infectivity in pDC-LDHB^−/−^ mice. Finally, we did not observe any reduction in CD86 or MHC-II expression in LDHB^−/−^ vs. WT pDCs, and in fact, MHC-II appeared to be increased in pDCs from pDC-LDHB^−/−^ mixed BM chimeras (Supplementary Fig. 4d). This small increase is, however, difficult to disentangle from the higher viral titers observed in pDC-LDHB^−/−^ mice (Fig. 3g), but generally supports that LDHB is not necessary for pDCs expression of antigen presentation molecules after infection. In contrast, our data show that LDHB expression in pDCs is essential for their optimal IFN-I production and early viral control after a beta coronavirus infection.

### LDHB is essential to human pDC function and downregulated in HIV infected patients

**Fig. 4:**
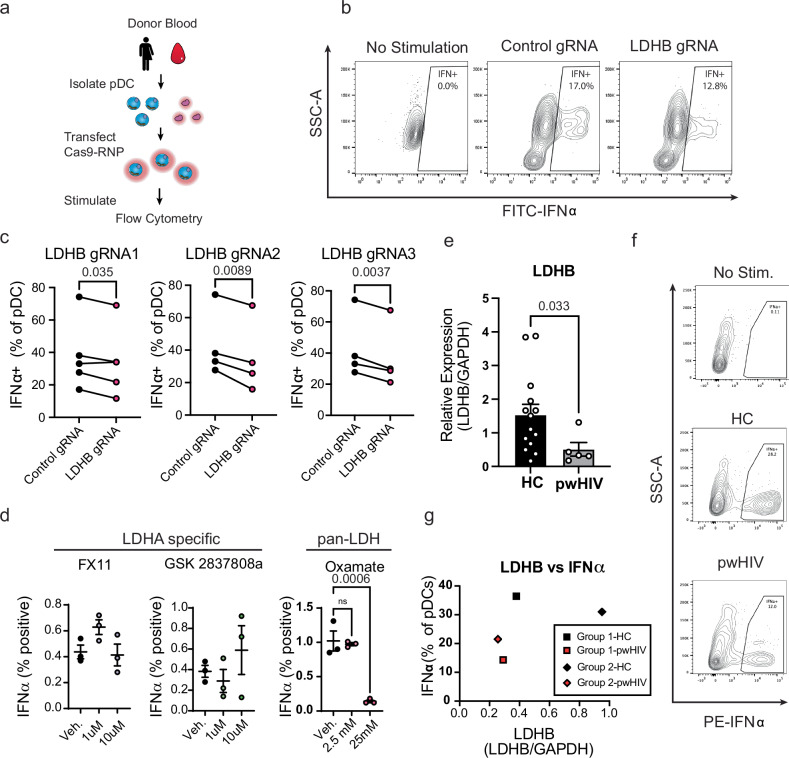
Human pDCs require LDHB for optimal IFN-I production. **a** Experimental outline for the modulation of gene expression in primary human pDCs. Briefly, PBMCs were isolated from donor blood, and pDCs were purified, then transfected with Cas9-RNP containing non-targeting control or targeting LDHB. 24 h later pDCs were stimulated with R848 and cytokine production measured by flow cytometry. **b** Representative plot of IFNα expression in Cas9-RNP transfected human pDCs after stimulation with R848. **c** IFNα production in Cas9-RNP transfected human pDCs. **d** Human PBMC were isolated from healthy donors, treated with the indicated small molecule inhibitors for 2 h prior and concurrent with stimulation, and IFNα was measured in pDCs by flow cytometry 8 h after stimulation with CpG-A. **e** qPCR quantification of LDHB transcripts in pDCs isolated from healthy controls (HC) or people with HIV (pwHIV). **f** Representative plot of IFNα staining from unstimulated HC (up), and HC, pwHIV stimulated with HSV-1 (middle and bottom). **g** comparison of LDHB expression and percentage of IFNα producing pDCs in two pairs (group 1 and 2) of HC and pwHIV processed in parallel. Data are representative of 3 (**d** left), 4 (**c** middle, **c** right, **d** middle, **d** right), or 5 (**c** left) independent experiments with unique donors, pooled averages from 19 patients quantified across 3 independent experiments (**e**), or 2 groups of age/sex matched HC and pwHIV (**g**). Data are shown as mean ± SEM. Statistics used are Two tailed Paired *t*-tests (**c**), Two tailed Mann-Whitney Test (**e**) or One-Way ANOVA with Dunnett correction (**d**).

Our above experiments describe a role for LDHB in the support of optimal IFN-I production in mouse pDCs. However, there are species-specific differences in pDCs from humans and mice^74^. Thus, we assessed whether LDHB was necessary for human pDC IFN-I production. For this, we made use of Cas9 ribonucleoprotein (RNP) technology. Briefly, RNP were assembled with a fluorescently tagged structural RNA and a crRNA targeting LDHB or a non-targeting control crRNA. These complexes were transfected into purified human pDCs; 24 h later these pDCs were stimulated with R848 and analyzed for cytokine production (Fig. 4a–c). Analysis of genome editing by T7 assay revealed low but readily detectable (~14–33%) editing (Supplementary Fig. 5a) in line with our results showing modest but significantly reduced LDHB protein expression by flow cytometry (Supplementary Fig. 5b, c). Our results showed that pDCs receiving LDHB targeting gRNAs produced less IFNα than those receiving control gRNA (Fig. 4b, c), but notably had no deficiency in TNFα production (Supplementary Fig. 5d) in line with our results from genetically deficient mice (Supplementary Fig. 3j) suggesting that genetic deficiency in LDHB specifically reduces IFN-I production.

To coordinately validate these results with a pharmacological approach we made use of available LDH inhibitors. As an LDHB specific inhibitor with validated capacity to inhibit LDHB activity in vivo has not been reported, we made use of Oxamate (OA) an LDH inhibitor which targets both LDHA and LDHB equally^75,76^ and contrasted this with inhibitors specific for LDHA (GSK2837808a^77^, or FX11^78^). We treated at a variety of concentrations within or below previously described EC50 ranges for each drug (GSK2837808a,11 0.4–11μM^77,79^; FX11, 49–60μM^80^; OA, 45-76mM^81^) and including the highest tested concentration that did not affect pDC viability (Supplementary Fig. 5e). Treatment with the selective LDHA specific inhibitors GSK2837808a or FX11 had no impact on IFN-I production in pDC from healthy donors after stimulation with CpG-A (Fig. 4d). In contrast, OA treatment severely reduced pDC capacity for IFN-I production (Fig. 4d) without compromising pDC viability (Supplementary Fig. 5e). TNFα production was compromised after OA treatment, suggesting a requirement of LDH for TNFα production in this case (Supplementary Fig. 5f). Similarly, MHC-II expression was reduced with OA treatment (Supplementary Fig. 5g). Notably, these results match what has been previously reported for GSK2837808a^60^ and suggest that activity of LDHA, but not LDHB, is dispensable for optimal human pDC IFN-I production after CpG-A stimulation.

Like mice, human pDCs show reduced capacity to produce IFN-I in the face of chronic viral infections such as HIV^82–85^ HCV^86–90^, and HBV^91–93^. To determine if LDHB downregulation is a conserved feature of suppressed pDC from protracted infections we investigated the expression of LDHB in pDCs from people with HIV (pwHIV) who have significantly reduced numbers of pDCs^82–84,94–96^. Purity of isolated pDCs was not different across groups (Supplementary Fig. 5h, Supplementary Fig. 6), with total purity ranging from 90 to 99% (Supplementary Fig. 5h, Supplementary Fig. 6, Supplementary Table 4). In agreement with our data from mice, LDHB expression was significantly decreased in pDCs from the peripheral blood of pwHIV specifically those with viremia (Fig. 4e). We also investigated whether LDHB expression related to the function of pDCs from pwHIV compared to healthy volunteers. Due to the significantly reduced numbers of pDCs in pwHIV^82,94,95^, we were only able to make this comparison for two pairs of donors. Comparison of stimulation of pwHIV and age/sex matched healthy controls revealed that these two patients were deficient in both LDHB expression and IFN production compared to their counterparts (Fig. 4f, g). These data are in line with a model in which LDHB expression and IFN-I production are both suppressed in pwHIV. However, it is important to interpret these findings cautiously given the limited number of samples in this study.

Altogether our results using genetic deletion, chemical inhibition, and investigation of LDHB in pwHIV support the assertion that LDHB contributes to optimal production of IFN-I by human pDCs, and LDHB downregulation is a conserved hallmark of suppressed pDCs in both humans and mice undergoing a sustained viral infection.

### pDCs and cDC2 show distinct metabolite preferences, and LDHB is essential to maintain optimal pDC metabolic capacity

**Fig. 5:**
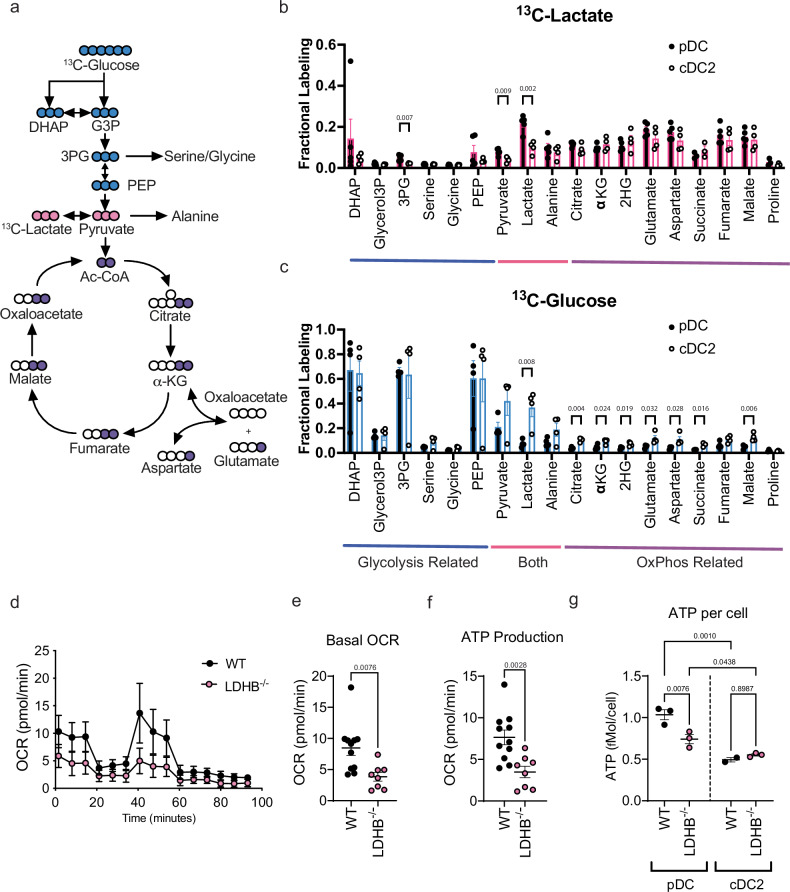
Both glucose and lactate feed the TCA cycle in DCs but pDCs are less labeled from glucose. **a** Diagram of stable-isotope tracing incorporation of ^13^C-Lactate and ^13^C-Glucose into glycolytic and TCA intermediates in pDCs isolated from Flt3L culture by FACS at day 8 p.c. Fractional labeling of glycolysis and TCA cycle intermediates in pDCs and cDC2 by ^13^C-Lactate (**b**) and ^13^C-Glucose (**c**) glycolysis and TCA cycle related metabolites are indicated below the graphs. **d** Seahorse tracing of WT (black) or LDHB^−/−^ (pink) pDCs. Derivative measures from Seahorse assay of WT (black) or LDHB^−/−^ (pink) pDCs (**e**) Basal OCR (**f**) ATP production. **g** Total ATP levels in WT (black) or LDHB^−/−^ (pink) pDCs or cDC2. Data are pooled from 2 (**b**, **c**), 3 (**d**), or 4 (**e**, **f**) independent experiments or representative of 3 independent experiments (**g**). Data are shown as mean ± SEM. Statistics used are Two tailed Student’s *t*-tests (**b**, **c**, **e**, **f**) or Two-Way ANOVA with Tukey Correction (**g**).

Given that LDHB is uniquely expressed in pDCs we hypothesized that there may be differences in energetic carbon sources used by pDCs as compared to conventional DCs. To investigate this, we generated pDCs or cDC2 by Flt3L culture, purified these cells by FACS and incubated them with either ^13^C labeled lactate, or ^13^C labeled glucose then measured incorporation into glycolysis and TCA related metabolites (Fig. 5a). Unexpectedly, we found that pDCs and cDCs similarly incorporated ^13^C from exogenously added lactate into their TCA cycle related metabolites (Fig. 5b). However, we observed a significantly reduced proportion of pDC TCA metabolites derived from glucose (Fig. 5c), suggesting that pDCs make less use of glucose (relative to lactate) to fuel their TCA cycles than cDC2 do comparatively under these conditions (Fig. 5c). Interestingly, concurrent stimulation from the initiation of culture with CpG did not alter lactate or glucose usage by either cDC2 or pDCs (Supplementary Fig. 7a–d).

We then attempted to determine if LDHB deficiency in pDCs modified their metabolic capacity. Indeed, we found that LDHB deficient pDCs showed significantly reduced basal OCR, and ATP production (Fig. 5d–g), but not reduced basal glycolysis (as defined by ECAR) (Supplementary Fig. 7e, f), or SRC (Supplementary Fig. 7g). Concurrently, we observed that LDHB deficient pDCs but not cDC2 showed reduced levels of ATP (Fig. 5g), in line with our observations that suppressed pDCs exhibit low ATP content (Fig. 2i). Overall, these data characterize the differential carbon source usage of lactate and glucose in pDCs versus cDC2s, and demonstrate that pDCs and cDC2s can both use significant amounts of lactate and glucose as carbon sources to fuel their oxidative metabolism, and LDHB deficient pDCs show significant defects in oxidative metabolism and ATP content.

### Restoration of LDHB rescues suppressed pDCs from infected mice

**Fig. 6:**
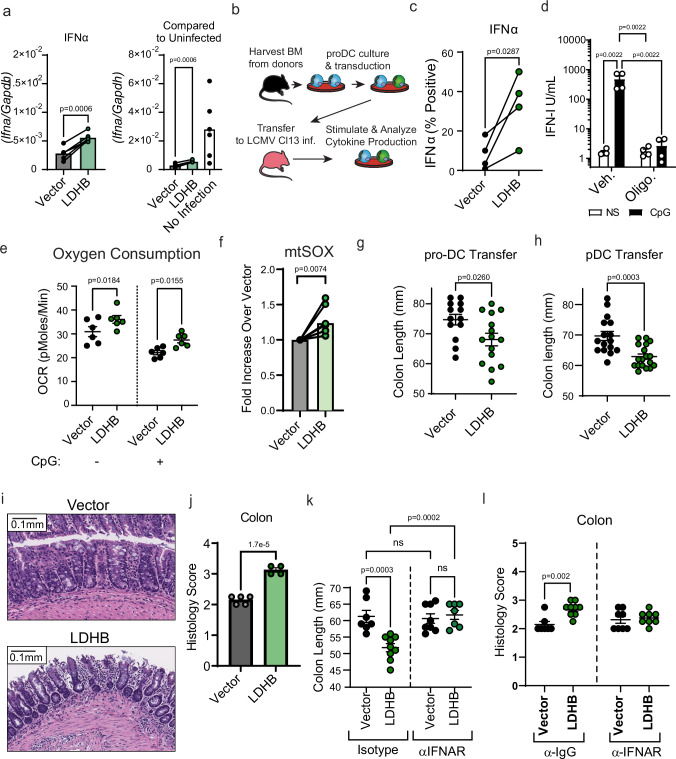
Enforced LDHB expression in suppressed pDCs restores function, and associates with potentiated infection-induced pathology. **a** Bone marrow from LCMV Cl13 infected mice at day 30 p.i. was isolated and subjected to Flt3L culture. Cultures from individual mice were separated and half-each transduced with retrovirus encoding LDHB or vector control. At day 8 p.c., pDCs were isolated by FACS and stimulated for 12 h with CpG and *Ifna* transcript was analyzed by qPCR. **b** Outline of proDC transfer experimental design to analyze pDC functional restoration in vivo. Bone marrow from uninfected mice was subjected to Flt3L culture and transduced with retrovirus encoding LDHB or vector control. At day 3.5 p.c. pro-DC were isolated by FACS and transferred into LCMV Cl13 infected mice at day 7.5 p.i. 6 days later splenocytes were isolated from recipients, and subjected to stimulation for 8 h with CpG-A, then interferon production in pDCs was assessed by flow cytometry. **c** IFNα production in pDCs derived from pro-DCs with enforced expression of vector control or LDHB was measured by flow cytometry. **d** pDCs isolated from the spleens of non-infected mice were treated with 1 µM Oligomycin for 2 h then stimulated with CpG for 6 h and IFN-I was measured by bioassay. Flt3L-culture-derived pDCs from day-30 LCMV Cl13 infected mice, expressing vector control or LDHB, were analyzed for OCR via Seahorse Xfe96 prior to or 30 min after stimulation with CpG (**e**) or mitochondrial derived superoxide (**f**). **g** Colon length was measured in LCMV Cl13 infected mice transferred with pro-DCs with enforced expression of vector control or LDHB, as depicted in b, at day 14 p.i. (day 6 post transfer). **h** Colon length was measured in LCMV Cl13 infected mice transferred with differentiated Flt3L-derived pDCs, with enforced expression of vector control or LDHB, at day 11 p.i. (day 2.5 post transfer). Representative (**i**) and quantitative (**j**) analysis of colon histology from LCMV Cl13 infected mice receiving differentiated pDCs as described in (**h**). Colon length (**k**) and histology score (**l**) were depicted as described in g for mice receiving differentiated pDCs and concurrently treated with either Isotype control antibody or IFNAR blocking antibody. Measurements were made blinded (**g**, **h**, **j**, **k**, **l**). Data are representative of 2 (**i**, **j**) experiments or pooled from 2 (a, d, e, k, l), 4 (**c**, **f**, **g**), or 5 (**h**) independent experiments. Data are shown as mean ± SEM. Statistics used were Two tailed Paired *t*-test (**a**, **c**, **f**) One-way ANOVA with Tukey Correction (**d**), Two tailed Student’s *t*-test (**e**, **g**, **j**), or two-way ANOVA with Fisher’s LSD (**k**, **l**) *p* < 0.05 *, *p* < 0.01; **, *p* < 0.001 ***, *p* < 0.0001 ****.

Given the essential role for LDHB in optimal pDC IFN-I production combined with its downregulation in dysfunctional pDCs from infected mice and humans, we then sought to determine if restoration of LDHB to suppressed pDCs could rescue their function. For this, we harvested BM cells from mice at day 30 p.i. and transduced them with a vector expressing LDHB in combination with enhanced (e)GFP or vector control at day 2 & 3 post-culture (p.c.) with Flt3L. At day 8 p.c., we stimulated cells with CpG-A and analyzed cytokine expression in transduced pDCs. We observed a significant increase in IFN-I production in LDHB transduced cells relative to those transduced with vector control, though this was at the lower end of the range of levels of IFN-I produced by pDC derived from the BM of uninfected animals (Fig. 6a). Along with our data indicating that LDHB deficiency does not completely ablate pDC IFN-I production or change TNFα production capacity these data reinforce the idea that multiple mechanisms are likely at play in the enforcement of pDC loss of function after infection. Still, it is important to note that this is the first identified method that can restore any amount of suppressed pDC function.

To further investigate whether LDHB restoration recovers pDC cytokine production in vivo, we made use of a newly developed system of pro-DC transfer. Briefly, in vitro developed pro-DCs, which can generate cDCs & pDCs^97^, were transduced to modulate gene expression, then transferred in vivo to complete their development into mature DCs (Fig. 6b). BM cells from uninfected mice were cultured with Flt3L and transduced at days 2–3 p.c. pro-DCs were then isolated at day 3.5 p.c. and transferred into LCMV Cl13 infected mice at day 7.5 p.i. Six days later (day 13.5 p.i.) we isolated splenocytes from these mice, stimulated them with CpG-A, and assessed the IFNα production of pDCs developed from the transferred pro-DCs by flow cytometry. We found consistently that LDHB overexpressing pDCs indeed had greater production of IFNα after isolation from infected mice (Fig. 6c). Together these data demonstrate that LDHB restoration can promote IFN-I production capacity in suppressed pDCs in vitro and in vivo.

### pDCs require oxidative metabolism for optimal IFN-I production and LDHB restoration improves oxidative metabolism in suppressed pDCs

It has recently been shown that oxidative metabolism is essential to human pDC function^60^. To directly assess whether this is also true in mice we isolated pDCs from uninfected animals, and pretreated them with Oligomycin (an inhibitor of ATP synthase), then stimulated them with CpG and measured IFN-I production by bioassay (Fig. 6d). This treatment completely ablated pDC IFN-I production, demonstrating that like human pDCs, mouse pDCs require oxidative metabolism for their production of IFN-I. We then investigated how LDHB restoration might improve the sustained metabolic deficiencies observed in dysfunctional pDCs from infected mice. For that, we first measured oxygen consumption in suppressed pDCs expressing LDHB or vector control, generated as in Fig. 6a. We found that pDCs expressing LDHB showed greater levels of oxygen consumption, and better sustained oxygen consumption following stimulation with CpG (Fig. 6e). In line with these data, expression of LDHB in pDCs derived from infected mice associated with significantly greater quantities of mtSOX, indicating that LDHB enforcement may improve oxidative metabolism in dysfunctional pDCs (Fig. 6f). Altogether these results suggest that pDCs require oxidative metabolism for IFN-I production, and enforcing LDHB expression in suppressed pDCs can improve oxygen consumption.

### LDHB expression in pDCs enhances virus-associated pathology

Despite exposing hosts to increased risk of secondary infection, pDC loss of function appears to be conserved across LCMV infection in mice^18,19,30^, SIV infection in macaques^98,99^, and humans infected with HIV^82–85^, HCV^86–89,100^, HBV^91–93^ or SARS-CoV-2^101^. This conservation suggests that pDC inhibition is an important feature of pDC biology, but its purpose is unknown. One salient but untested hypothesis is that pDC suppression, like CD8 T cell exhaustion^102^, exists to protect the host from overt immune driven pathology. To investigate this, we returned to the proDC transfer model that allowed pDC functional restoration (Fig. 6b, c). LCMV Cl13 infection causes colitis-like symptoms including colon shortening^103^, additionally in non-infectious conditions, release of pDC negative regulators has been established to drive intestinal pathology^104^. Therefore, we focused our investigation on the intestinal compartment of these mice. Intriguingly, in 4 of 4 experiments we observed shortening of the colons for mice receiving LDHB expressing proDCs as compared to their vector transferred counterparts (Fig. 6g). Notably, while IFN-I is critical for many viral infections^8^ treatment with recombinant IFNα is not impactful for viral replication at later stages after LCMV Cl13 infection^105^. Accordingly, we observed only a mild reduction in viral titers that did not reach significance when transferring LDHB expressing proDCs in LCMV Cl13 infected mice (Supplementary Fig. 8a).

It is important to recognize that proDCs can differentiate into both cDC1 and cDC2 as well as pDC. Although we did not observe any alterations to cDC proportion, cDC function, or DC maturation (as measured by expression of CD86) in the populations derived from LDHB expressing proDCs after transfer (Supplementary Fig. 8b–d), suggesting that cDCs are similar in both conditions, these experiments did not directly demonstrate a relationship between LDHB restoration in pDC and colitis. Thus, to determine whether the enhanced colon shortening was pDC dependent, we performed similar experiments transferring mature pDCs derived from uninfected mice via Flt3L culture and transduced to express LDHB or vector control into LCMV Cl13 infected mice at day 8.5 p.i. and investigating colon length 2.5 days later at day 11 p.i. As expected, mice receiving LDHB expressing pDCs exhibited shorter colons than their vector control counterparts (Fig. 6h). Furthermore, histological examination found increased inflammation in these same colons (Fig. 6i, j) confirming that transfer of LDHB expressing pDCs into infected mice associated with increased colitis. Intriguingly, we observed no significant increase in pathology in the small intestine or liver of these animals (Supplementary Fig. 8e, f), suggesting that some tissues may be more susceptible to pathology caused by LDHB-expressing pDCs during viral infection.

We then sought to determine whether this increased inflammation was the result of IFN-I production. To this end, we repeated the experiments as described, but provided mice transferred with vector or LDHB with either IFNAR blocking antibody^106^ or isotype control antibody immediately following the transfer of vector or LDHB expressing pDCs, and again at days 0.5 and 1.5 post-transfer. The mice treated with isotype control antibody recapitulated what we observed in untreated mice, showing shorter colons in the group transferred with LDHB expressing pDCs versus their vehicle control group (Fig. 6k). In contrast, treatment with anti-IFNAR resulted in full restoration of colon length in the mice with LDHB expressing pDCs (Fig. 6k), and eliminated the difference in both colon length and inflammatory score by histology between animals transferred with LDHB-expressing or vector control pDCs (Fig. 6k, l). Altogether these results demonstrate that LDHB expression in suppressed pDCs restores their function, and that during in vivo viral infection, the presence of pDCs that resist functional loss was accompanied with increased IFN-I dependent colitis. This suggests that LDHB expression and pDC inhibition manage a trade-off between supporting pDC function and opposing host-pathology during viral infection.

## Discussion

Type I IFN is a critical antiviral and antineoplastic cytokine. As pDCs produce the largest quantities and most types of IFN-I of any cell type, they are critical first responders for many viral infections^12^. Accordingly, pDC suppression associates with increased susceptibility to secondary infection^18,19,82^. Until now there has been no method to restore this downregulated pDC IFN production, and so there has been no tool available to understand the physiological consequences of ablating this well conserved process^18,19,82–88,90–93,98,99^. Our work has established that pDC suppression is associated with profound metabolic deficiencies. While various environmental factors may contribute to the observed pDC metabolic alterations, in this study we focused on LDHB downregulation, a shared phenotype across different anatomical compartments, time points post-infection, viruses, and species. Notably, our results revealed that LDHB supports the metabolism of pDCs as well as the production of IFN-I by pDCs in both humans and mice. We also showed that LDHB expression in pDCs was downregulated by persistent infections and that restoration of LDHB reinstated IFN-I production capacity in the otherwise dysfunctional pDCs in vitro and in vivo. Furthermore, this allowed us, for the first time, to determine the consequence of restoring function to suppressed pDCs. In the context of persistent LCMV infection, enforced LDHB expression enhanced virus-induced-colitis^103^ in an IFNAR dependent manner, suggesting that pDC inhibition may be a conserved mechanism which provides protection against excessive pathology during viral infection.

Through our analysis we generated the first gene signature of TLR-dependent pDC exhaustion. Notably, this independently identified several known regulators of pDC IFN-I production (*Cd81*^55,56^*, Myc*^57^) as well as several genes with unknown relationship to pDC function, but previously identified as differentially expressed in pDC from SIV-infected macaques (*Tigit*)^54^. Tigit is of particular interest since it is a surface expressed protein and a known regulator of exhaustion in CD8 T cells that is being explored as a target in cancer immunotherapy^107^. We also reconfirmed our previous observation that suppressed pDCs from infected mice acquire a proliferative phenotype^30^. While this may appear controversial, this is also a key phenotype of exhausted T cells, which include a highly proliferative stem-like subset^108–110^.

Our study identified *Ldhb* as a “pDC exhaustion signature” gene that is downregulated after infection while in uninfected mice was uniquely highly expressed in pDCs as compared to other DCs. This may be because LDHB is a target of E2-2 (encoded by *Tcf4*), an essential transcriptional regulator of the pDC lineage^111,112^ which we have previously shown to be downregulated in suppressed pDCs from both humans and mice^30^. It is thus tempting to speculate that reduced levels of E2-2 drive LDHB downregulation in dysfunctional pDCs, however further work will be needed to support this hypothesis.

By identifying LDHB as an essential regulator of pDC IFN-I production, our study adds to a significant body of work by other groups understanding essential metabolic regulators of pDC function at steady state, or shortly following stimulation^58–60,113,114^. Our research does much to reinforce general principals discovered by these groups, including an essential role for oxidative metabolism in pDC IFN-I production^58–60,113^. Importantly, our study is the first to investigate how pDC metabolism changes in vivo following infection, and how these metabolic adaptations relate to pDC loss of function. It is important to address that it was previously reported that LDH activity is dispensable for human pDC IFN-I production^60^, the inhibitor used in this case (GSK2837808a) has high specificity for LDHA, and so this is still in line with our observation that human and mouse pDCs rely on LDHB, but not LDHA, for IFN-I production. Indeed, we were able to recapitulate the fact that GSK2837808a does not impact pDC IFN-I production after CpG-A simulation, reinforcing the dispensability of LDHA specifically in regulating pDC IFN-I production (Fig. 4d).

Our work also demonstrates for the first time that both pDCs and cDCs can use extracellular lactate as a significant carbon source for TCA cycle metabolites on par with glucose. This is a potentially important observation as, in the context of inflammatory environments, where other cells such as activated macrophages, T cells, or tumor cells (in the case of a tumor microenvironment) consume large quantities of glucose and excrete large quantities of lactate, DCs may need to rely on lactate to support oxidative metabolism. Further work will be needed to pursue this hypothesis, as well as to determine the preferred carbon sources of these cells in supporting their TCA cycles including assessment of glutamate and fatty acid oxidation.

Very little is understood about pDC IFN-I inhibition in the context of cancer, but it has been described across a variety of types of malignancies^20–23^, suggesting the phenomenon is at least somewhat conserved. Adjuvant therapies that stimulate pDCs to produce IFN-I have shown great promise in the treatment of some varieties of cancer^24–27^, and our study suggests that coordinated metabolic and stimulatory intervention may be more successful than either approach alone. On the other hand, our work also provides insight into what types of therapies may inadvertently compromise pDC activity. A generalized LDH inhibition, for example, has been proposed as a mechanism to treat highly glycolytic cancers. Our study suggests that inhibitors which do not target LDHB may be more effective as they are likely to avoid further suppressing pDC function.

Given the well-established anti-viral and antitumor roles of IFN-I^8,9^, it has previously been unclear why pDC suppression is conserved across species. Our study provides one possible reason in that we observe more severe virus associated pathology in animals where pDC loss of function is opposed. In addition, following an infection, it may also be beneficial to default to the active suppression of IFN-I production as there are several bacterial infections, including ancient infections (e.g., *Mycobacterium tuberculosis, Listeria monocytogenes*, *Staphylococcus aureus*), for which IFN-I responses can be detrimental^115–118^. Furthermore, as most viruses encode highly effective interferon evasion strategies^29^ continued production of IFN-I after establishment of infection may have diminishing returns for viral control. Indeed, treatment with recombinant IFN-β and IFN-α5 at late stages of LCMV Cl13 infection did not affect viral titers^105^ and persistent interferon signaling counterintuitively leads to CD8 T cell exhaustion and promotes viral persistence^119,120^. Conversely, early application of IFN-β and IFN-α5 in the same infection model leads to improved viral control^105^, therefore delaying pDC suppression might be expected to improve viral control, although the relative tradeoff in immunopathology in this case is not known. Interestingly, pDC functional impairment has been described in systemic lupus erythematosus (SLE), a fact sometimes used to assert their lack of importance for this disease^121^. However, it is possible that pDC inhibition emerges after transient IFN-I production (as in the viral infection), and that de novo generated pDCs may continuously provide a new source of IFN-I prior to becoming suppressed. Additionally, pDC loss of function in these patients may be incomplete.

Altogether, our study characterizes the transcriptional state of pDCs throughout viral infection, and as a result identifies large scale metabolic reprogramming in suppressed pDCs. We identify LDHB as a novel regulator of human and mouse pDC IFN-I production which is downregulated during pDC loss of function and demonstrates that restoring LDHB rescues IFN-I production capacity to suppressed pDCs. This is associated with enhanced IFNAR dependent infection-induced-colitis when LDHB expressing pDCs are transferred into infected mice, suggesting the conservation of pDC inhibition after infection may be a mechanism to avoid excessive pathology. These data provide the first proof-of-concept showing that it is possible to restore suppressed pDC function, and the first evidence that the purpose of conservation for pDC inhibition across species is to protect against immunopathology. Furthermore, our study provides insights into the metabolic machinations pDCs use to maintain optimal IFN-I production, and concurrently identify novel avenues to manipulate pDC function for the benefit of human health.

## Methods

### Mice

Six to eight-week-old female C57BL/6 (Strain #000664), B6 CD45.1 (Strain #002014), sham-operated and ADX mice were purchased from The Jackson Laboratory. TLR7^−/−^ mice (Strain #008380) and BDCA2-DTR mice (Strain #014176) were purchased from The Jackson laboratory and bred in house. LDHB^−/−^ mice (C57BL/6NTac-Ldhb^tm1a(KOMP)Wtsi^) were generated by the international mouse phenotyping consortium (IMPC, www.mousephenotype.org, https://www.mousephenotype.org/data/genes/MGI:96763) and procured from the Knockout Mouse Consortium (KOMP). All mice were housed under specific-pathogen-free conditions at the University of California, San Diego at an ambient temperature of 22-25 C and ambient humidity with a 12 h day-night cycle. For all experiments experimental and control animals were cohoused up until the point of infection, at which point infected and uninfected groups were kept in separate cages. When two infected groups were compared, they were housed separately through the course of infection. For pDC depletion mixed BM chimeras of 50% BDCA2-DTR and 50% either WT or LDHB^−/−^ BM were treated with DT (Sigma-Aldrich, Catalog #:D0564)^70^ Mice were given 200 ng of DT by intraperitoneal injection daily starting from 2 days prior to MHV infection, and up to endpoint (48 h p.i.). Mice were euthanized via CO_2_ chamber per the recommendations of the UCSD Institutional Animal Care and Use Committee (IACUC). Mouse handling and experiments conformed to the requirements of the National Institute of Health and the IACUC Guidelines of UC San Diego. Permission was granted to perform experiments as described by the UC San Diego IACUC. Unless stated otherwise, experiments were initiated in mice (female and male) at 7–12 weeks of age.

### Virus strains

LCMV Infection was performed by injecting mice with 2 × 10^6^ plaque-forming units (pfu) of LCMV Cl13^122^ intravenously (i.v.) via the tail vein. LCMV Cl13 was propagated in BHK cells and quantified by plaque assay performed on Vero cells^122^ (ATCC CCL-81) Vero cell monolayers were infected with serially diluted viral stock and incubated for 60 min at 37 °C in 5% CO_2_ with gentle shaking every 15 min. SeaKem ME Agarose (Lonza, Catalog #: 50011) overlay was added to infected cells and placed in an incubator for 6 days at 37 °C in 5% CO_2_. Cells were fixed with formaldehyde (Sigma, F7503) and stained with crystal violet (Sigma-Aldrich, Catalog #:C0775-25G) for 5 min at room temperature and plaques were counted. MHV A59 was a generous gift from Prof. Susan Weiss^123^. MHV infection was performed by injecting 50 p.f.u./mouse intraperitoneal (i.p.). MHV titers were assessed as described above for LCMV^124^, using L929 cells (ATCC: CCL-1) and incubating with agar overlays for 2 days prior to formaldehyde fixation.

### Human samples inclusion and ethics

All human subject studies were approved by the Institutional Review Board at Rutgers, the State University of New Jersey, New Jersey Medical School. Blood samples from HIV-negative and HIV-infected volunteers were obtained with consent according to institutional guidelines and the Declaration of Helsinki.

### Human pDC isolation

Human pDC isolation was performed using an EasySep Human Plasmacytoid DC Enrichment kit (STEMCELL Technologies, Catalog #:19062) following the manufacturer’s instructions.

### Cas9 RNP generation and transfection into human pDCs

Alt-R® Cas9-guides were predesigned by integrated DNA technologies (IDT), sequences reported in Supplementary Table 5. Cas9 RNP were generated using Alt-R S.p. HiFi Cas9 Nuclease (IDT, Catalog #: 1081061), Alt-R CRISPR-Cas9 tracrRNA ATTO (IDT, Catalog #: 1075928), in combination with control crRNA (IDT, Catalog #: 1072544) or crRNA targeting LDHB (IDT, Supplementary Table 5). Human pDCs were isolated as above and cultured 16 h in RPMI-1640 (Gibco Catalog #: 21870084) with the addition of 10% (vol/vol) Fetal Calf Serum (FCS), 2mM L-Glutamine (Gibco, Catalog #: 25030081) and 20 ng/mL of recombinant human IL-3 (R&D systems, Catalog #: 203-IL-010). Cas9 and gRNA components were assembled into RNP per the manufacturer’s instructions and transfected into pDC 16 h p.c. using Lipofectamine CRISPRMAX transfection reagent (Invitrogen, Catalog #: CMAX00015) per the manufacturer’s instructions. Cells were then cultured for an additional 24 h in the above-described conditions prior to analysis of function.

### Flt3L DC culture

Flt3L DC culture from infected and uninfected mice was performed as in our previous publications^30,125^. Bone marrow was isolated from mice, and BM DCs were generated at the concentration of 2 × 10^6^ cells/ml for 8 days in 5 ml of DC medium (RPMI-1640 (Gibco Catalog #: 21870084) supplemented with 10% (vol/vol) FCS, L-glutamine (Gibco, Catalog #: 25030081), penicillin-streptomycin (Cytiva, Catalog #: SV30010), and HEPES pH 7.2 (Fisher Scientific, Catalog #:15630080)) 100 ng/mL Flt3L (provided by CellDex Therapeutics) and 50 µM β-mercaptoethanol (Gibco, Catalog #: 21985023). Half of the medium was replaced after 5 days with medium with fresh Flt3L added.

### Generation of retroviral particles (RV) and retroviral transduction

HEK293T cells (ATCC CRL-3216) were transfected with plasmid encoding LDHB or vector only control, LT1 transfection reagent (Mirus Bio, Catalog #: MIR 2304), and the pCL-ECO packaging plasmid^126^ (Addgene Plasmid #12371). Supernatants were harvested 48 h later and kept at 4 °C until media were replaced with fresh media and the second supernatants were harvested 24 h after media change (72 h post-transfection). Fresh supernatants containing RV without any freezing were used for retroviral transduction. At day 2 post-Flt3L culture, BM cells were transduced with RV encoding vector control or LDHB and 10ug/ml polybrene reagent (Tocris Biosciences, Catalog #: 7711) and spin-infected at room temperature at 1000 × *g* for 90 min. Cells were incubated 37˚C overnight. On day 3 post-Flt3L culture, cells were again transduced with RV encoding vector control or LDHB. Cells were then washed in PBS twice and placed in fresh DC medium + Flt3L.

### Cell sorting

Spleens were incubated with 1 mg/mL collagenase D (Sigma-Aldrich Catalog #:11088882001) for 20 min at 37 °C and pushed through a 100μm strainer to make a single cell suspension. Mouse bones (femur and tibia) were isolated, cleaned of any muscle tissue, then flushed with RPMI-1640 (Gibco Catalog #: 21870084) with the addition of 10% (vol/vol) Fetal Calf Serum (FCS), 2mM L-Glutamine (Gibco, Catalog #: 25030081), penicillin-streptomycin (Cytiva, Catalog #: SV30010), and HEPES pH 7.2 (Fisher Scientific, Catalog #:15630080), to retrieve BM. Splenocytes or BM cells were then subjected to red blood cell lysis in 1 mL ammonium-chloride-potassium lysis buffer for 5 min. Splenocytes or BM cells were enriched using EasySep Mouse Streptavidin RapidSpheres Isolation Kit (Stemcell Technologies Catalog #: 19860) per the manufacturer’s instructions. Biotin conjugated anti-Thy1.2 (53-2.1, eBioscience Catalog #: 13-0902-82) and anti-CD19 (eBio1D3, eBioscience Catalog #: 13-0193-82). Cells were stained with propidium iodide (PI) (Sigma Aldrich Catalog #: P4864) as well as streptavidin conjugated to PerCP-Cy5.5 (eBioscience, 45-4317) (to account for any biotin-conjugated antibody remaining post enrichment) and stained for flow cytometry prior to FACS-purification of pDCs by the following definitions: pDC; PI^-^, CD19^-^, Thy1.2^-^, NK1.1^-^, CD11c^+^, CD11b^-^, B220^+^, BST2^+^, cDC1; PI^-^ CD19^-^, Thy1.2^-^, NK1.1^-^, CD11c^+^, CD11b^-^, B220^-^, CD8a^+^, cDC1; PI^-^, CD19^-^, Thy1.2^-^, NK1.1^-^, CD11c^+^, CD11b^+^, B220^-^. Where Flt3L culture was used pDCs were harvested from culture and stained with PI by the following definition: PI^-^, CD11c^+^, CD11b^-^, B220^+^, BST2^+^. proDC^97^ were FACS purified at day 3.5 after Flt3L culture by the following definition: PI^-^, CD19^-^, B220^-^, Thy1.2^-^, CD3^-^,CD4^-^,CD8^-^,CD127^-^, NK1.1^-^,Ter119^-^,Gr-1^-^,MHC-II^-^, CD11c^-^, CD11b^-/low^. All sorting was performed on a BD ARIA II flow cytometer (BD Biosciences). Antibodies manufacture clones and fluorophores for each of the antibodies used for flow cytometry can be found in Supplementary Table 7.

### Cell transfer

For proDC and mature pDC transfer cells were FACS isolated at days 3.5 and 8 p.c. respectively following retroviral transduction as described above. For pro-DCs between 2 × 10^5^and 5 × 10^5^ cells were injected intravenously into recipients at day 7.5 p.i. in a total of 200 μL in PBS. For mature pDCs between 1 × 105 and 2 × 10^5^ cells were injected intravenously into recipients at day 8.5 p.i. and isolated 2.5 days later at day 11 p.i. For anti-IFNAR treatment mice were given anti-IFNAR antibody MAR-1 (Bio X Cell, Catalog #: BE0241) (500 μg/mouse) on the day of transfer, and 250 μg/mouse at days 0.5, and 1.5 post transfer by intraperitoneal injection.

### Metabolic flux assays

To analyze OCR and ECAR Seahorse Xfe96 plates (Agilent, Catalog #: 103794-100) or Seahorse XF HS Miniplates (Agilent, Catalog #:103725-100) and cassettes were prepared per the manufacturer’s instructions. On the day of the experiment 1.5 × 10^5^ pDCs from the BM (primary pDC) or 500,000 pDCs (Flt3L Culture) were isolated by FACS into RPMI 1640 Seahorse Assay Medium (Agilent, Catalog #: 103576-100) with the addition of 10% FCS, 2mM L-Glutamine (Agilent, Catalog #: 103579-100) & 10 mM Glucose (Agilent, Catalog # 103577-100), prior to plating onto Poly-L-Lysine coated 96 well assay plates. Cells were then rested for 1 h at 37C without additional CO_2_. Assays were either performed with a Seahorse Xfe96 analyzer (Fig. 6e) or a XF HS Mini (All other figures). Mitochondrial stress test assay for pDC on the XF-HS Mini was optimized in-house within the ranges provided by the manufacturer and performed using the mitochondrial stress test kit (Agilent, Catalog #: 103010-100), with the addition of 2-DG (Millipore Sigma, Catalog #: D8375) in the 4th injector port. In well concentrations for each drug at time of injection were Oligomycin (1.5 μM), FCCP (2uM), Antimycin/Rotenone (0.5 μM), and 2-DG (50 mM). Basal OCR was calculated as the difference between OCR prior to any drug addition and OCR after Antimycin/Rotenone. SRC was calculated as the difference between OCR prior to the addition of any drug and the OCR measured after FCCP addition. ATP production was calculated as the difference between OCR prior to the addition of any drug and the OCR measured after Oligomycin addition. Basal glycolysis was calculated as the difference between ECAR prior to any drug addition and ECAR after the addition of 2-DG.

### ELISA & IFN bioassay

After FACS purification pDCs were stimulated with 1 µM CpG-A (Invivogen ODN 1585, Catalog #: tlrl-1585), and supernatant was collected for Bioassay or ELISA at 6 or 12 h respectively. IFNα was measured by Lumikine Xpress mIFNα ELISA 2.0 (InvivoGen, Catalog #: luex-mifnav2) following manufacturer instructions. IFN-I bioactivity was measured with reference to a recombinant mouse IFNβ standard (Sigma-Aldrich, Catalog #: IF011) using ISRE-L929 reporter cells (L-929 cell line transfected with an interferon-sensitive luciferase)^30^. For each assay limit of detection was determined as the lowest concentration used for the standard curve multiplied by the dilution factor of the samples, all unstimulated samples were below limit of detection. For stimulated samples below limit of detection values are reported at the limit of detection.

### qRT-PCR

Isolated immune subsets were subjected to RNA extraction using an Rneasy micro kit (Qiagen, Catalog #: 74004) including treatment with Dnase I (Qiagen, Catalog #: 79254) to remove genomic DNA. RNA was reverse transcribed into cDNA using Superscript III RT (Invitrogen, Catalog #: 12574026). The expression of various genes was quantified using Fast SYBR Green Master Mix (Thermo Fisher Scientific, Catalog #: 4385618) or TaqMan Fast Universal PCR Master mix (Thermo Fisher Scientific, Catalog #: 4352042) with probe sets from the Universal Probe Library (Roche). The CFX96 Touch Real-Time PCR Detection System (Bio-Rad) was used to quantify reactions. Primers for the indicated genes are listed in Supplementary Table 6. Relative transcript levels were normalized against mouse *Gapdh* or human *GAPDH*^30^. Detectable range for each primer set used was determined by evaluating the linear range of a series of dilutions. CT values above detectable range were considered to be non-detected, and their values set as maximum cycle number (45 cycles).

### Flow cytometry

Antibodies manufacture clones and fluorophores for each of the antibodies used for flow cytometry can be found in Supplementary Table 7, and example gating in Supplementary Fig. 9. The following antibodies were used to stain single cell suspensions prepared from murine BM or spleens or mouse Flt3L culture: Thy 1.2 (53-2.1, eBioscience), CD19 (eBio1D3, eBioscience), NK 1.1 (PK136, eBioscience), Gr-1 (RB6-8C5, BioLegend), CD11c (N418, eBioscience), CD11b (M1/70, BD Biosciences), B220 (RA36B2, BioLegend), BST2 (eBio927, eBioscience), CD8 (53-6.7, eBioscience), CD45.1 (A20, eBioscience), CD45.2 (104, eBioscience), MHC class II (I-A/I-E) (M5/114.15.2, BioLegend), CD86 (GL1, eBioscience), Ter-119 (TER-119, eBioscience), CD127 (A7R34, eBioscience), CD3 (145-2C11, eBioscience), CD4 (RM4-5, eBioscience), CD8 (53-6.7, eBioscience). The following antibodies were used to stain human pDCs either from PBMCs or after purification and culture as described in the text: CD3 (UCHT1, BioLegend), CD14 (HCD14, BioLegend), CD16 (3G8, BioLegend), anti-human CD19 (HIB19, BioLegend), anti-human CD56 (MEM-188, BioLegend), HLA-DR (L243, BioLegend), CD11c (B-ly6, BD Biosciences), CD123 (6H6, BioLegend), CD304 (12C2, BioLegend), LDHB (EP1566Y, abcam) TNF-α (Mab11, BioLegend), IFN-α (LT27:295, Miltenyi Biotec). PI (Sigma Aldrich, Catalog #: P4864) or Ghost dye (Tonbo Biosciences, Catalog #: 13-0863-T100) was used to exclude dead cells. Cells were pre-incubated with CD16/CD32 Fc block (BD Biosciences, Catalog #: 553141) for 10 min prior to surface staining. Mitochondrial dyes (MitoSOX Red Thermo Fisher Catalog #: M36008, MitoTracker Red chloromethyl-X-rosamine (CMXRos) Thermo Fisher Catalog #: M46752, MitoTracker Green Catalog #:FM M46750) were used per the manufacturer’s instructions. Cells were acquired with a ZE5 flow cytometer (Biorad) and data were analyzed using FlowJo software (BD Biosciences).

### Isotopic tracing gas chromatograph-mass spectrometry (GC-MS) sample preparation and analysis

pDCs were isolated by FACS sorting as described above, then rested for 1 h in DC medium (see formulation above). Labeled ([3-^13^C]-Lactate, Cambridge Isotope Laboratories Catalog #: CLM-1578) or unlabeled lactate (Sigma-Aldrich) and labeled (U-^13^C6, Cambridge Isotope Laboratories Catalog #: CLM-1396-1) or unlabeled glucose (Thermo Fisher Scientific, Catalog #: 15023021) were then added to generate a culture with a final concentration of 5 mM Lactate and 10 mM glucose, and incubated for 3 h. Afterward cells were collected, washed in cold 0.9% NaCl, then frozen and stored at −80 °C prior to extraction. Metabolites were extracted, analyzed, and quantified^127^. Mass isotopomer distributions and total metabolite abundances were computed as in ref. ^127^, labeling is depicted as the fraction of labeled metabolites as in ref. ^127^.

### Histopathological analysis

Colon, SI and Liver samples were isolated from mice, fixed, embedded, sectioned, and stained using hematoxylin/eosin^128^ by the Tissue Technology Shared Resource at UCSD. Scoring of colon and SI inflammation was performed using the inflammation score defined in Erben et al. 2014^129^. Scoring of Liver was performed by applied pathology systems using the method described in reference^130^.

### Statistics and reproducibility

Randomization and blinding were used in the measurement of colon length, as well as the scoring of colon inflammation where the measurer was blind to the treatment of the mice. All relevant data are shown as mean ± standard error. The number of animals or samples are stated in figure legends. Specific statistical tests are stated in figure legends. We used Student’s *t*-test when comparing two groups where samples were independently generated, or Paired *t*-test when samples were derived from the same animal. In cases where variances were unequal, as defined by significant F test, unpaired *t* test with Welch’s correction was used. Multiple comparisons corrections were used when more than one comparison was performed and are stated in legends. One way ANOVA with Tukey correction was used when there were many independent comparisons. All analyses were performed with Graphpad Prism 10 software.

### Reporting summary

Further information on research design is available in the Nature Portfolio Reporting Summary linked to this article.

## Supplementary information

Supplementary Information

Description of Additional Supplementary Files

Supplementary Data 1

Supplementary Data 2

Supplementary Data 3

Supplementary Data 4

Supplementary Data 5

Supplementary Data 6

Reporting Summary

Peer review file

## Source data

Source data

## Data Availability

The RNAseq and Microarray data have been deposited in the Gene Expression Omnibus (GEO) database under GSE285244 and GSE285874 at. Other data and reagents are available from the corresponding author on request. All data are included in the Supplementary Information or available from the authors, as are unique reagents used in this Article. The raw numbers for charts and graphs are available in the Source Data file whenever possible. Source data are provided with this paper.

## Code availability

All software used to perform these analyses is publicly available. Software tools are listed in the main text and methods.
