# Supplementary material for: Metabolic deficiencies underlie reduced plasmacytoid dendritic cell IFN-I production following viral infection
Source: Nat Commun. 2025 Feb 7;16:1460. doi: 10.1038/s41467-025-56603-5 (PMC11805920; doi:10.1038/s41467-025-56603-5)
Supplement: Supplementary file 1 — Supplementary Information [file 41467_2025_56603_MOESM1_ESM.pdf]

Metabolic Deficiencies Underlie Reduced Plasmacytoid Dendritic Cell IFN-I Production following Viral Infection

Supplementary Information

First Author: Trever T. Greene
Corresponding Author: Elina I. Zuniga

Supplementary Figure 1

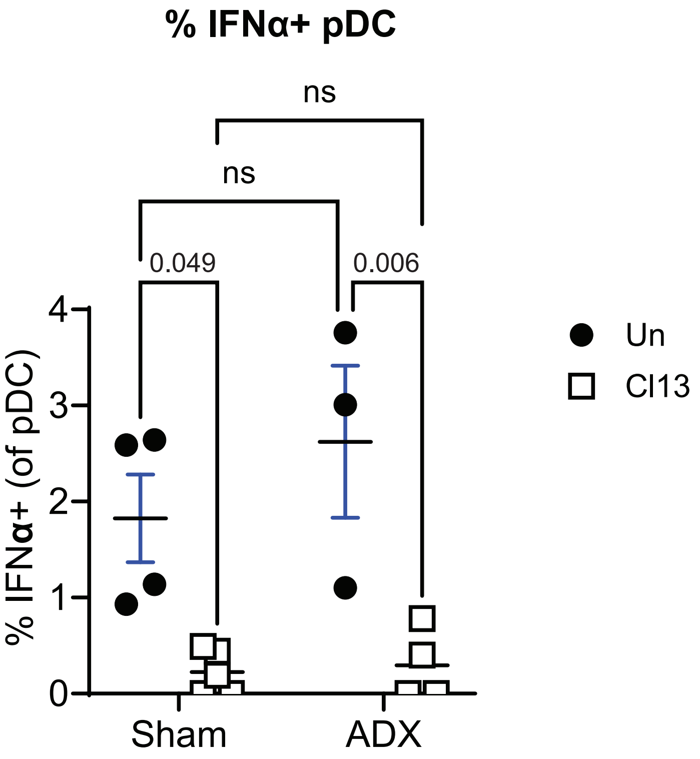

Supplementary Data Figure 1. Glucocorticoids do not drive loss of IFN-I production in pDCs. Sham-operated (Sham) or adrenalectomized (ADX) C57BL6/J mice were left uninfected or infected with LCMV Cl13. At day 4 p.i., splenocytes were stimulated with CpG-A, and IFNα production analyzed by flow cytometry. Data are shown as mean ± SEM. Data are representative of 2 independent experiments. Statistical significance was determined by two-way ANOVA with Tukey correction for multiple comparisons.

Supplementary Figure 2

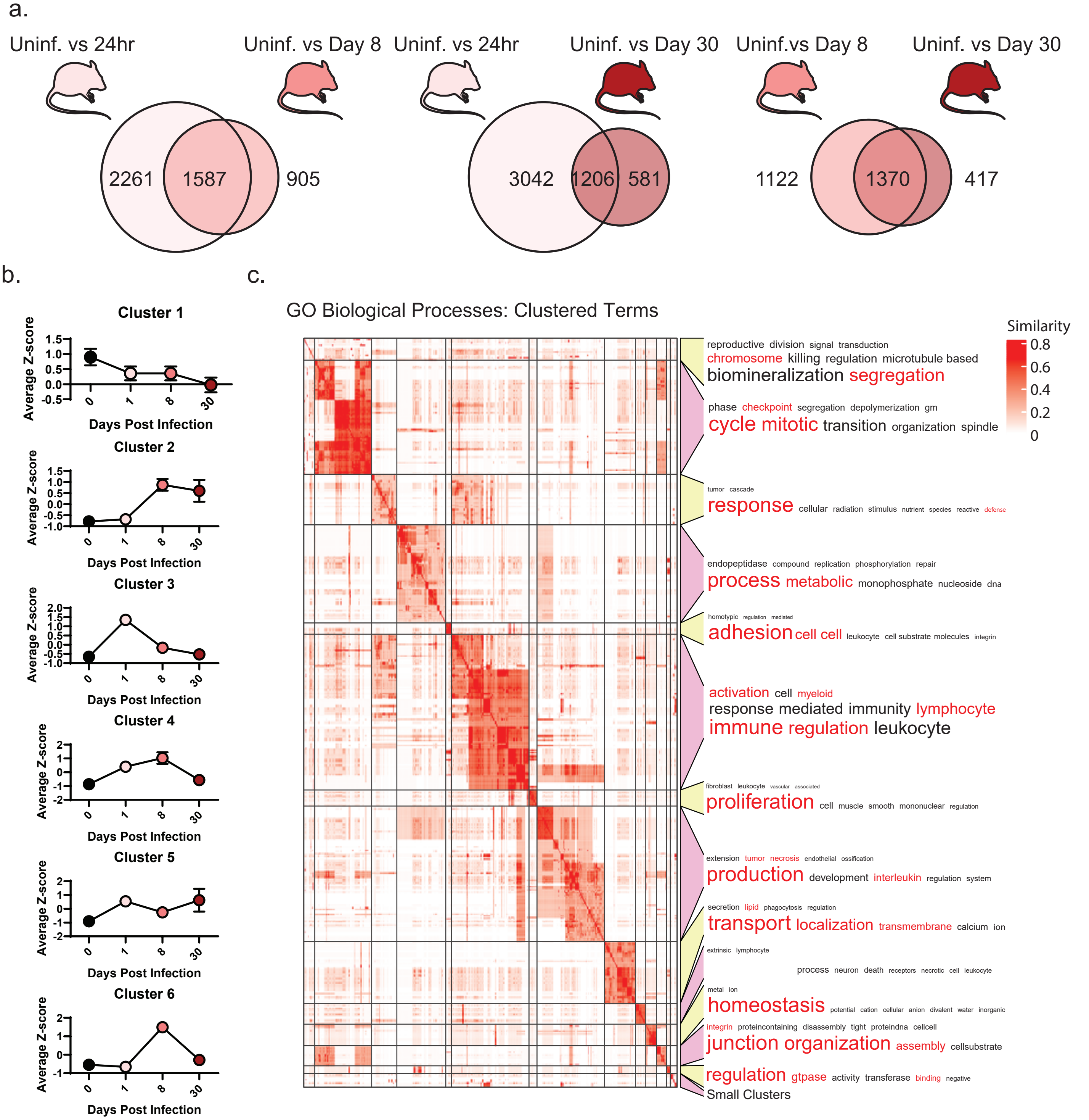

Supplementary Data Figure 2. Transcriptional analysis of pDCs throughout persistent viral infection. (a) Number of DE or overlapping genes between pDCs isolated from uninfected mice (Uninf.) or from mice at days 1 (24 hr), 8, or 30 p.i. (b) Average Z-score for expression of genes in each cluster (as identified in Fig. 1) at each timepoint. (c) GO Biological processes identified as enriched in genes differentially expressed both when comparing uninfected to day 8 p.i., as well as comparing uninfected to day 30 p.i. (pDC suppression related) were grouped by semantic similarity. Word clouds for each cluster identified by this analysis are presented with larger font representing higher frequency within the biological processes in that cluster.

Supplementary Figure 3

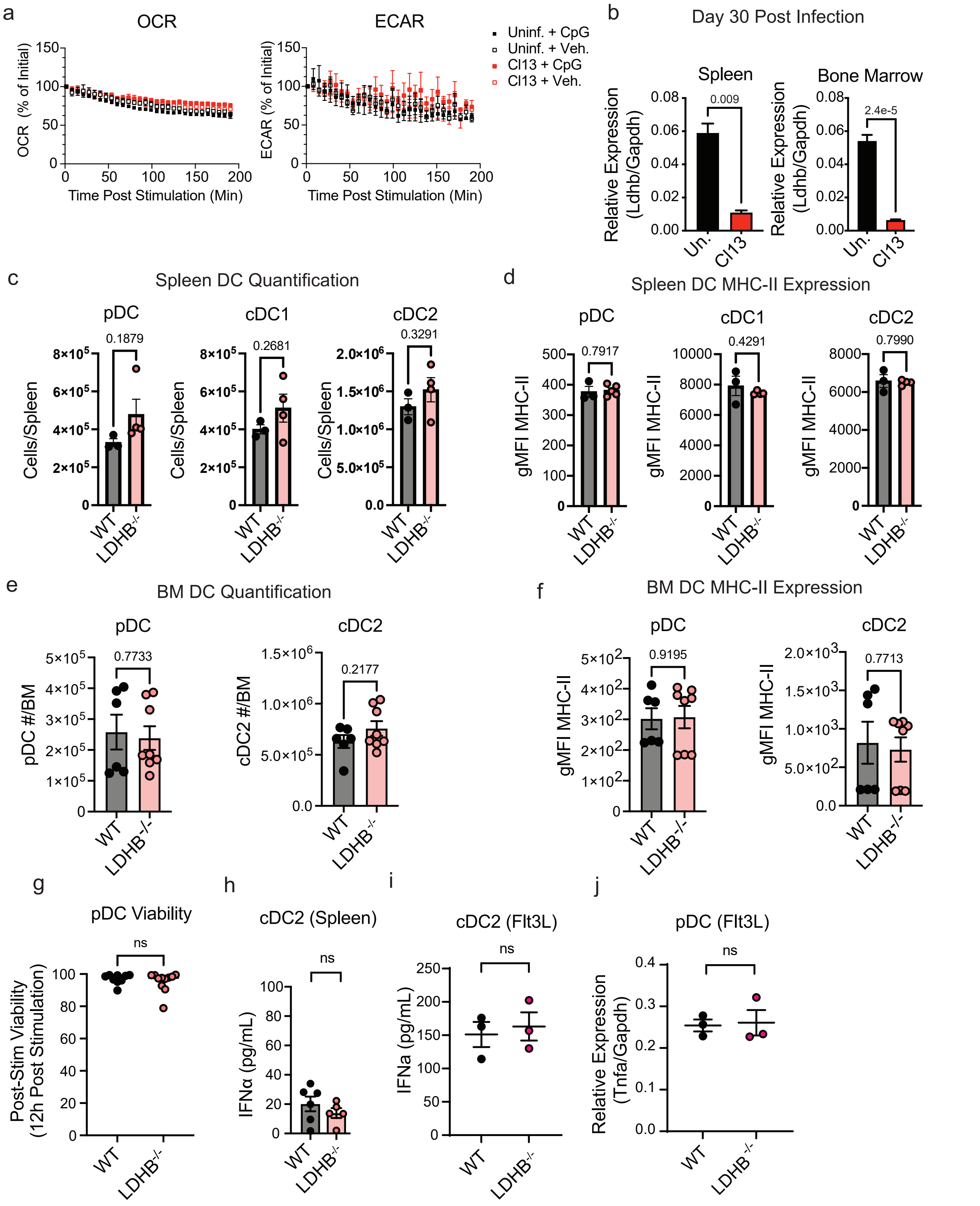

Supplementary Data Figure 3. pDCs modify metabolism and expression of LDHB in infection, and LDHB deficiency does not change DC numbers, MHC-II expression, viability or IFN production by cDC2. (a) OCR and ECAR traces of pDCs from uninfected mice (black) or LCMV Cl13 infected mice (red) at day 8 p.i. following stimulation with CpG (closed symbols), or no stimulation (open symbols). (b) qPCR analysis of Ldhb expression in pDCs isolated from the spleen or BM of mice at day 30 p.i. (c-f) Number of pDCs (c,e), and expression of MHC-II (d,f) were measured by flow cytometry in the spleen (c,d) and BM (e,f) from WT (black) or LDHB-/- (pink) mice. (g) FACS purified pDCs were stimulated with CpG-A for 12 hrs, supernatant collected for ELISA as in Fig. 3b, and viability was measured 12 hrs after CpG stimulation by flow cytometry. (h,i) ELISA of IFNα from cDC2 isolated from spleens (h) or Flt3L cultures (i) from WT (black) or LDHB-/- (pink) mice. (j) qPCR analysis of Tnfa transcript in Flt3L culture derived pDC stimulated with CpG-A for 12 hrs. Data are pooled from 2-3 (a,e,f,g,h) or representative of 2-4 independent experiments (b-d,i,j). Data are shown as mean ± SEM. Statistics used Student’s T Test (b-j).

Supplementary Figure 4

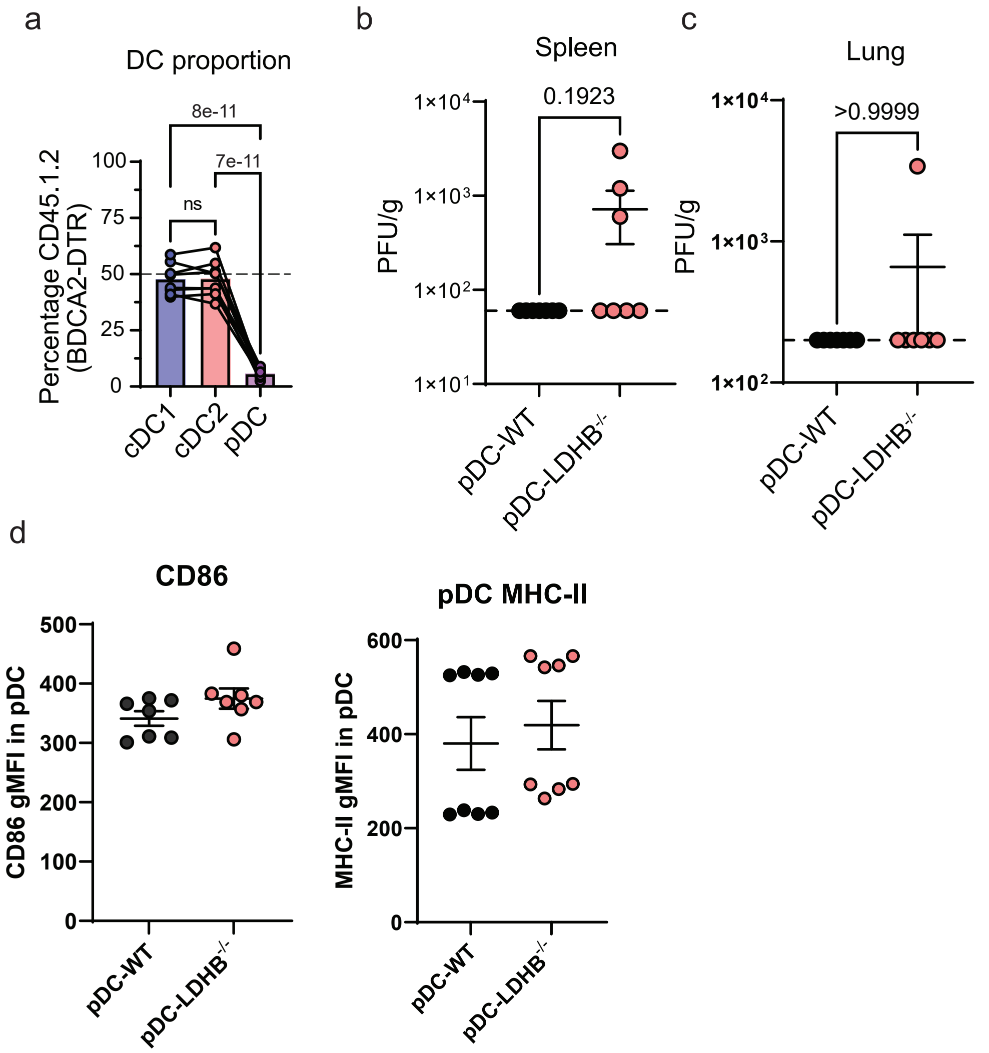

Supplementary Data Figure 4. BDCA2-DTR mixed bone marrow chimeras. (a) proportion of cDC1 (blue), cDC2 (red), and pDC (purple), from the CD45.1.2 BDCA2-DTR compartment of MHV infected mice described in Fig. 3e after treatment with DT at 48 h.p.i. (b,c) Plaque forming units of MHV from the Spleen (b) or Lung (c) of MHV infected mice described in Fig. 3e at 48 h.p.i. Limit of detection shown as dotted line, samples with no countable plaques are shown at the limit of detection. (d) CD86 and MHC-II expression in pDCs isolated from the spleens of the MHV infected mixed BM chimeras as described in Fig. 3e was measured by flow cytometry at 48 h.p.i. Data are pooled from 2-3 independent experiments (a-d). Data are shown as mean ± SEM. Statistics used are One Way ANOVA with Tukey Correction (a). Student's T Test (b-d).

Supplementary Figure 5

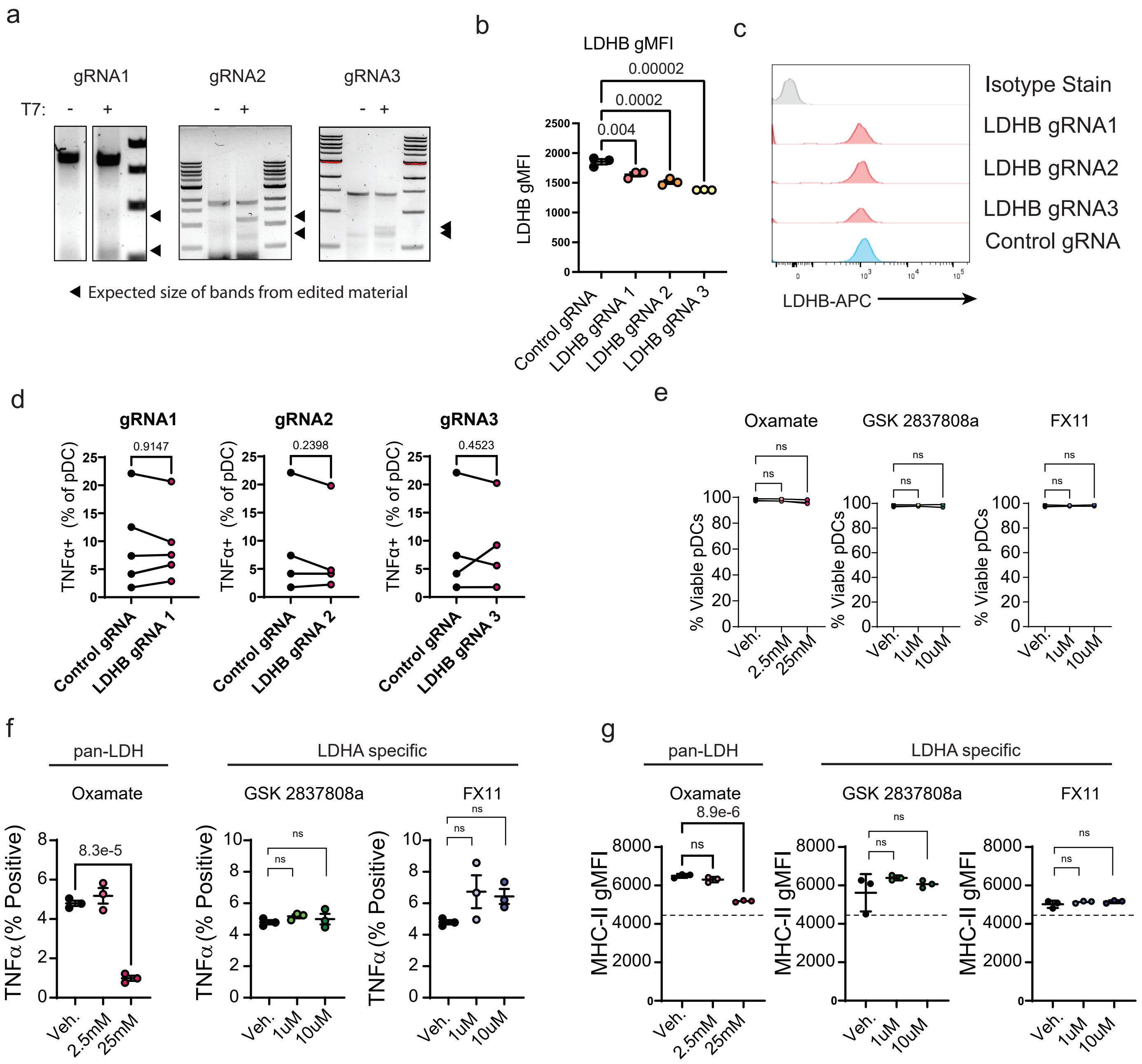

h

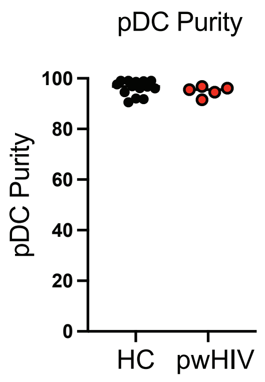

Supplementary Data Figure 5. Modulation of LDHB in human pDCs regulates their function. (a) Representative gel for T7 assays using DNA isolated from pDC transfected with LDHB targeting gRNA. Arrows represent expected sizes for bands indicating editing. (b) gMFI of LDHB in pDCs transfected with LDHB targeting gRNA was measured by flow cytometry. (c) Representative histograms from b. (d) TNFα production in human pDCs transfected with the indicated Cas9-RNPs after stimulation with R848 as in Fig. 4. (e) PBMCs were treated with Oxamate, GSK2837808a, or FX11, stimulated with CpG-A and pDC viability was measured by flow cytometry. (f,g) TNFα (f) or MHC-II (g) expression in pDCs from PBMCs treated with the indicated inhibitors and stimulated with CpG-A for 8 hrs. (h) purity of pDC isolated from HC or pwHIV determined by FACS after enrichment. Data are pooled from 3-5 individual donors (d-g), representative of 3-5 independent experiments (a-c,f,g), or pooled averages from 19 patients quantified across 3 independent experiments (h). Data are shown as mean ± SEM. Statistics used are One Way ANOVA with Tukey Correction (b,e-g) or Paired T Test (d) p<0.05 *, p<0.01; **, p<0.001 ***, p<0.0001 ****.

Supplementary Figure 6

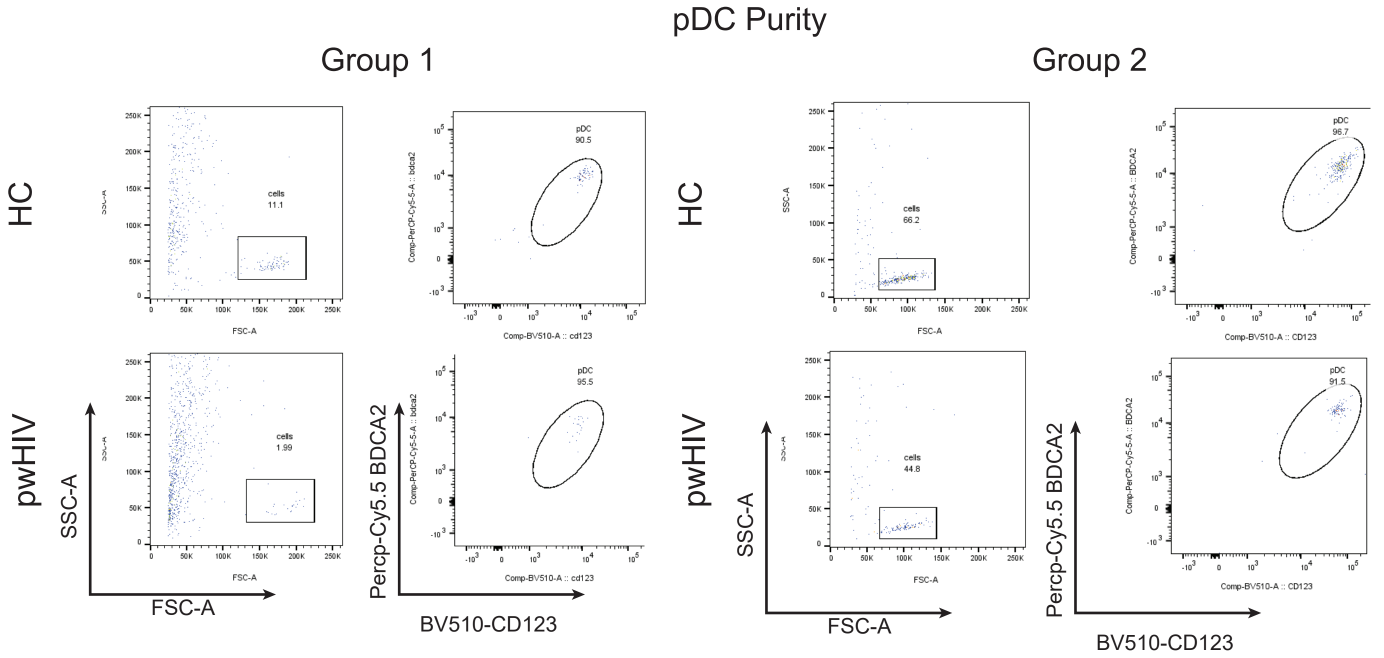

Supplementary Data Figure 6. Purification of pDC from pwHIV. Flow cytometry analysis of 1 purified pDC from healthy controls (HC) and people with HIV (pwHIV) for two groups of 2 age/sex matched donors, pDCs are defined as CD123+, BDCA2+.

Supplementary Figure 7

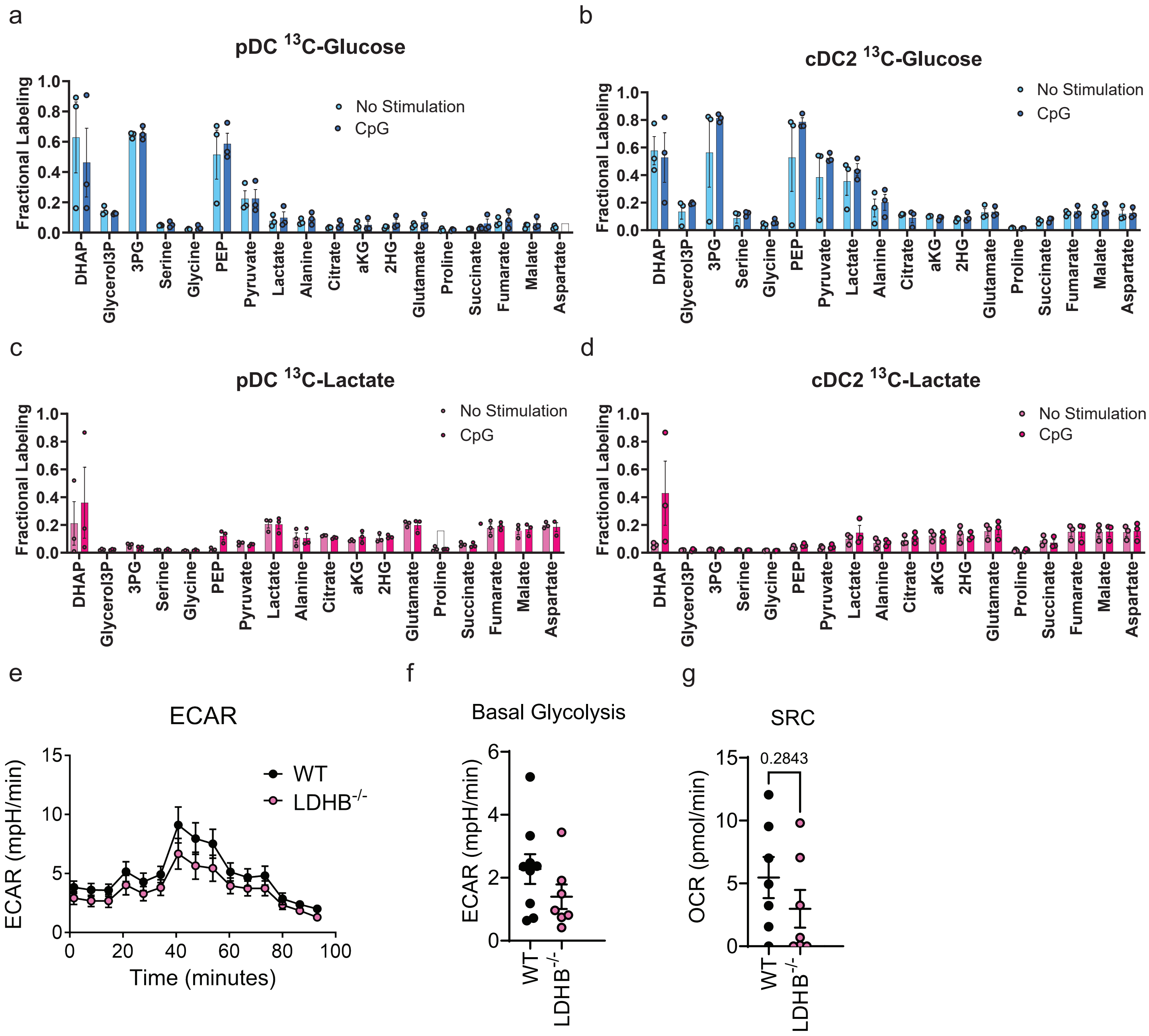

Supplementary Data Figure 7. Dendritic Cell Metabolic Profiling. Fractional labeling of glycolysis and TCA cycle intermediates by 13C-Glucose (a,b) 13C-Lactate (c,d) in pDCs (a,c) and cDC2 (b,d) isolated from Flt3L culture. During incubation with 13C-sources cells were either unstimulated (light), or stimulated with CpG-A (dark). (e) ECAR trace from seahorse of WT (black) and LDHB-/- (pink) pDCs. (f) Basal Glycolysis of pDCs from WT (black) and LDHB-/- (pink) mice. (g) Spare Respirator Capacity of pDCs from WT (black) and LDHB-/- (pink) mice. Data are pooled from 3-4 independent experiments (a-g). Data are shown as mean ± SEM. Statistics used are Student's t test (a-d,f,g).

Supplementary Figure 8

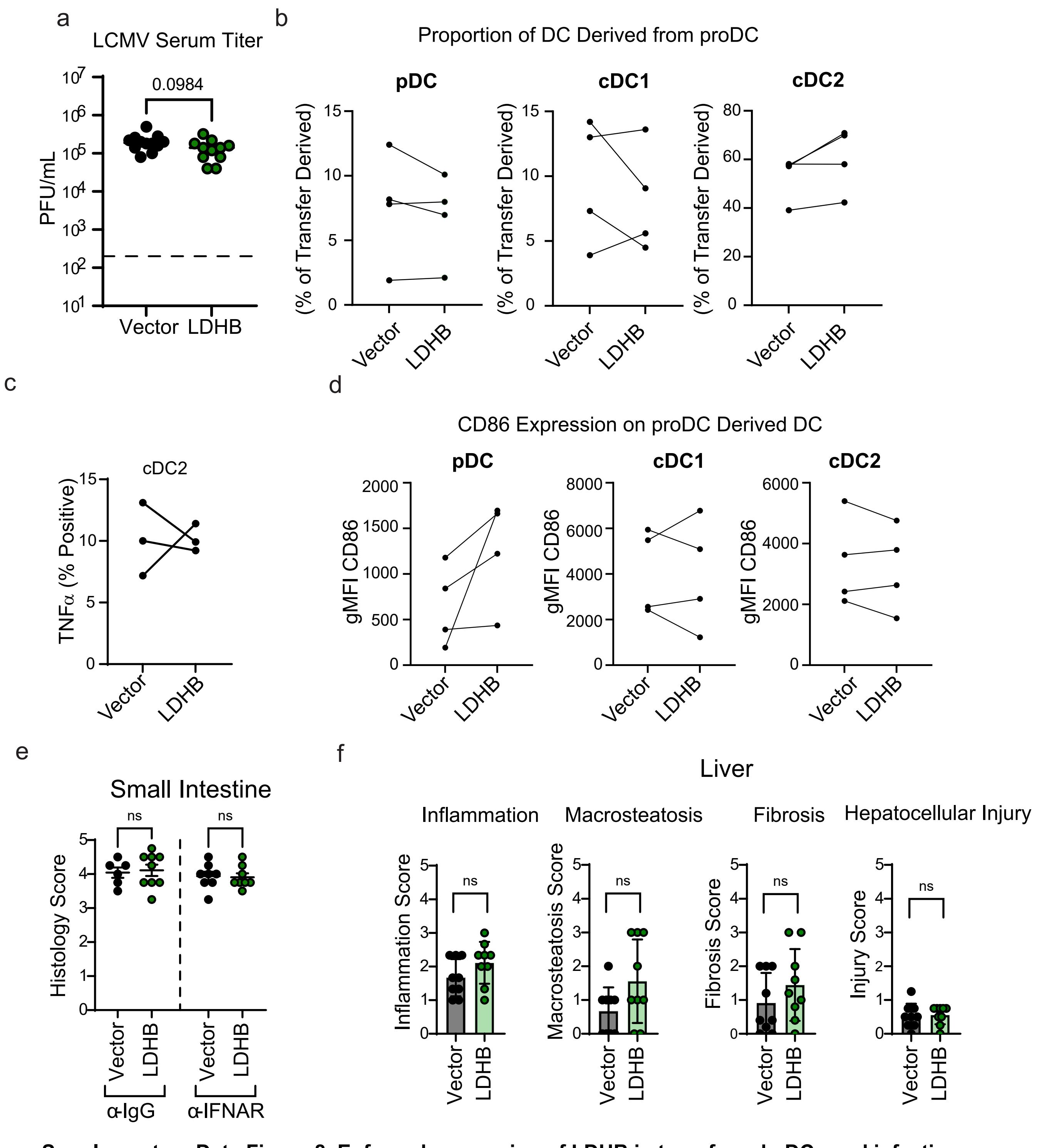

Supplementary Data Figure 8. Enforced expression of LDHB in transferred pDCs and infection outcome. (a) Viral titers measured by plaque assay in serum day 13.5 p.i. (6 days after transfer of vector or LDHB expressing pro-DCs), as described in Fig. 6b. (b) proportion of spleen DCs derived from vector or LDHB expressing pro-DCs after in vivo transfer was evaluated by flow cytometry. (c) TNFα production in cDC2 derived from vector or LDHB expressing pro-DC transferred into LCMV Cl13 infected animals after CpG-A stimulation for 8 hrs. (d) Expression of CD86 on pDC, cDC1, and cDC2 derived from vector or LDHB expressing pro-DCs after stimulation with CpG-A for 8 hrs. (e,f) Small intestine (e) and Liver (f) histology scores after transfer of differentiated pDCs expressing vector or LDHB as described in Fig. 6. Data are pooled from 2-4 experiments (a-f). Statistics used are student's t test (a,f), paired t test (b-d), and two-way ANOVA with Fisher's LSD test (e).

Supplementary Figure 9

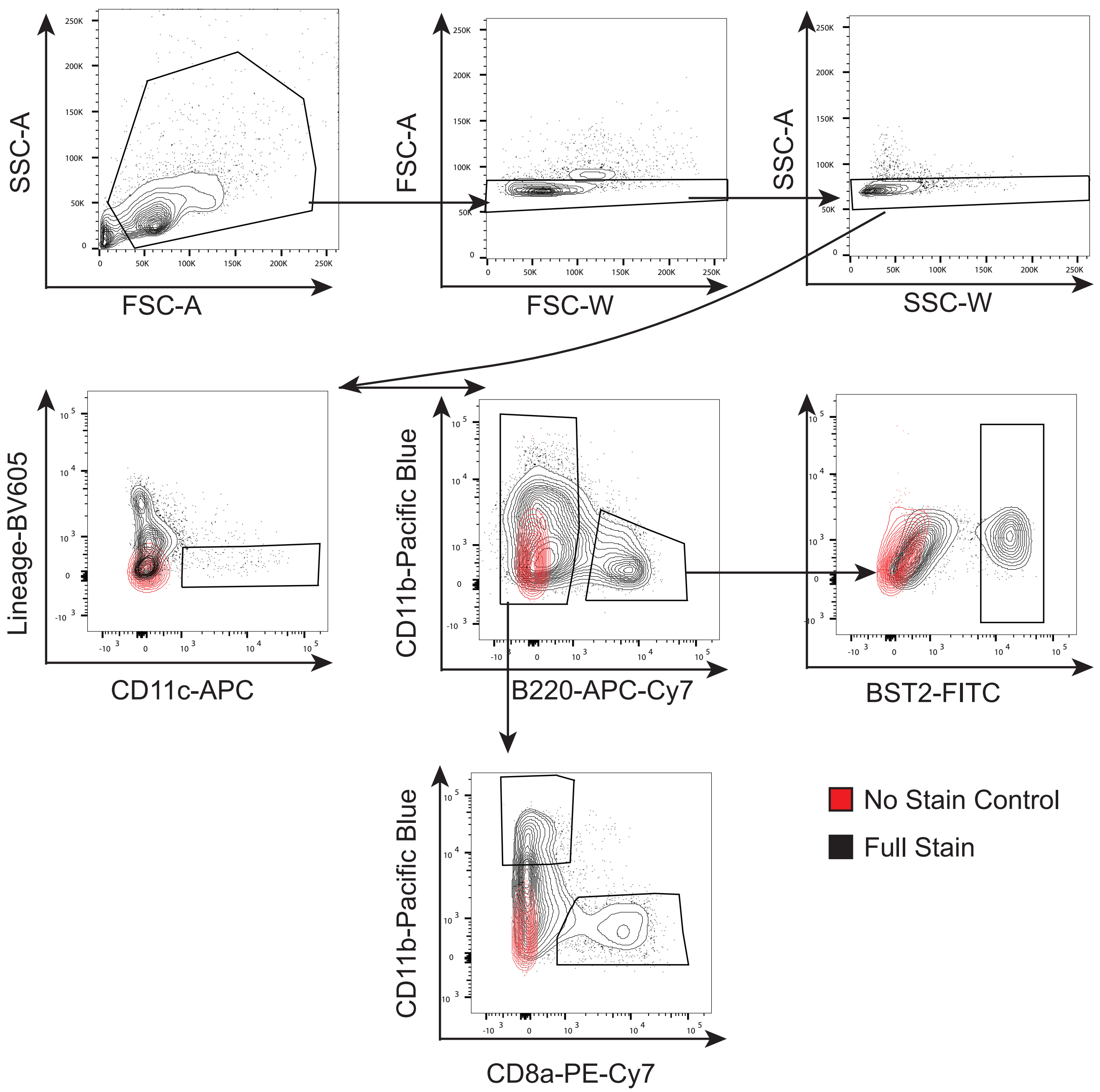

Supplementary Data Figure 9. Flow Cytometry Gating Strategy. Gating strategy for DCs is presented.

Supplementary Table 1: GSEA of 24hr infection vs NI

ID	Description	setSize	enrichmentScore	NES	pvalue	p.adjust	qvalue
mmu04060	Cytokine-cytokine receptor interaction - Mus musculus (house mouse)	240	0.650028652	1.670802324	1.00E-04	1.00E-04	0.001096221
mmu04080	Neuroactive ligand-receptor interaction - Mus musculus (house mouse)	237	0.587764184	1.510715867	1.00E-04	1.00E-04	0.001096221
mmu04151	PI3K-Akt signaling pathway - Mus musculus (house mouse)	329	0.583924569	1.508437564	1.00E-04	1.00E-04	0.001096221
mmu05165	Human papillomavirus infection - Mus musculus (house mouse)	330	0.578111687	1.493476734	1.00E-04	1.00E-04	0.001096221
mmu05163	Human cytomegalovirus infection - Mus musculus (house mouse)	234	0.53902745	1.385324075	1.00E-04	1.00E-04	0.001096221
mmu05200	Pathways in cancer - Mus musculus (house mouse)	504	0.514030841	1.334017035	1.00E-04	1.00E-04	0.001096221
mmu05171	Coronavirus disease - COVID-19 - Mus musculus (house mouse)	225	0.624949976	1.605147751	1.00E-04	1.00E-04	0.001096221
mmu05169	Epstein-Barr virus infection - Mus musculus (house mouse)	212	0.590291739	1.514699846	0.00010002	0.00010002	0.001096221
mmu05170	Human immunodeficiency virus 1 infection - Mus musculus (house mouse)	216	0.552458471	1.417986255	0.00010002	0.00010002	0.001096221
mmu05167	Kaposi sarcoma-associated herpesvirus infection - Mus musculus (house mouse)	204	0.563691336	1.445048421	0.00010003	0.00010003	0.001096221
mmu04621	NOD-like receptor signaling pathway - Mus musculus (house mouse)	176	0.632786467	1.618081024	0.00010004	0.00010004	0.001096221
mmu05164	Influenza A - Mus musculus (house mouse)	161	0.680584378	1.736539344	0.00010006	0.00010006	0.001096221
mmu05161	Hepatitis B - Mus musculus (house mouse)	159	0.587217451	1.497924727	0.00010008	0.00010008	0.001096221
mmu04630	JAK-STAT signaling pathway - Mus musculus (house mouse)	154	0.672089883	1.714154376	0.00010013	0.00010013	0.001096221
mmu05160	Hepatitis C - Mus musculus (house mouse)	153	0.667333069	1.701838648	0.00010014	0.00010014	0.001096221
mmu04514	Cell adhesion molecules - Mus musculus (house mouse)	152	0.609065999	1.552970085	0.00010014	0.00010014	0.001096221
mmu05162	Measles - Mus musculus (house mouse)	142	0.611278425	1.555619421	0.00010017	0.00010017	0.001096221
mmu04936	Alcoholic liver disease - Mus musculus (house mouse)	136	0.620897686	1.578487902	0.00010018	0.00010018	0.001096221
mmu04217	Necroptosis - Mus musculus (house mouse)	128	0.663621755	1.683977608	0.000100241	0.000100241	0.001096221
mmu04650	Natural killer cell mediated cytotoxicity - Mus musculus (house mouse)	113	0.634788311	1.605645531	0.000100392	0.000100392	0.001096221
mmu04620	Toll-like receptor signaling pathway - Mus musculus (house mouse)	100	0.735893829	1.854856564	0.000100543	0.000100543	0.001096221
mmu04623	Cytosolic DNA-sensing pathway - Mus musculus (house mouse)	72	0.758054025	1.883122599	0.000101678	0.000101678	0.001096221
mmu04622	RIG-I-like receptor signaling pathway - Mus musculus (house mouse)	68	0.794748758	1.96762783	0.000101999	0.000101999	0.001096221
mmu04721	Synaptic vesicle cycle - Mus musculus (house mouse)	67	0.665632822	1.64723964	0.000102062	0.000102062	0.001096221
mmu05320	Autoimmune thyroid disease - Mus musculus (house mouse)	61	0.713076068	1.753538276	0.000102501	0.000102501	0.001096221
mmu04974	Protein digestion and absorption - Mus musculus (house mouse)	87	0.604371079	1.514684863	0.000202	0.000202	0.001975561
mmu04610	Complement and coagulation cascades - Mus musculus (house mouse)	77	0.633704475	1.578975669	0.000202963	0.000202963	0.001975561
mmu04940	Type I diabetes mellitus - Mus musculus (house mouse)	53	0.670480094	1.631017849	0.000206889	0.000206889	0.001975561
mmu05417	Lipid and atherosclerosis - Mus musculus (house mouse)	203	0.538418748	1.380143302	0.00040012	0.00040012	0.00354128
mmu03260	Virion - Human immunodeficiency virus - Mus musculus (house mouse)	8	-0.940445446	-2.117843499	0.000407664	0.000407664	0.00354128
mmu05150	Staphylococcus aureus infection - Mus musculus (house mouse)	60	0.645291592	1.584607412	0.000410593	0.000410593	0.00354128
mmu05142	Chagas disease - Mus musculus (house mouse)	101	0.585808231	1.477042018	0.000502614	0.000502614	0.004199469
mmu05152	Tuberculosis - Mus musculus (house mouse)	174	0.543162574	1.388410991	0.0006003	0.0006003	0.004772192
mmu04512	ECM-receptor interaction - Mus musculus (house mouse)	83	0.599648289	1.499616097	0.000606857	0.000606857	0.004772192
mmu04933	AGE-RAGE signaling pathway in diabetic complications - Mus musculus (house mouse)	98	0.581780185	1.465629977	0.000704155	0.000704155	0.005379105
mmu04061	Viral protein interaction with cytokine and cytokine receptor - Mus musculus (house mouse)	79	0.602500067	1.503718458	0.000810373	0.000810373	0.00584265
mmu05323	Rheumatoid arthritis - Mus musculus (house mouse)	77	0.599487753	1.493719254	0.000811853	0.000811853	0.00584265
mmu05322	Systemic lupus erythematosus - Mus musculus (house mouse)	50	0.657379684	1.5930051	0.000830392	0.000830392	0.00584265
mmu05330	Allograft rejection - Mus musculus (house mouse)	48	0.648887457	1.566745115	0.001147507	0.001147507	0.007866848
mmu04024	cAMP signaling pathway - Mus musculus (house mouse)	195	0.526514749	1.348573919	0.00130052	0.00130052	0.008692951
mmu04727	GABAergic synapse - Mus musculus (house mouse)	81	0.582013584	1.45390748	0.00192483	0.00192483	0.012552167
mmu04020	Calcium signaling pathway - Mus musculus (house mouse)	218	0.50894959	1.306351949	0.00210042	0.00210042	0.013371095
mmu00100	Steroid biosynthesis - Mus musculus (house mouse)	19	-0.703426666	-2.086406022	0.002279635	0.002279635	0.014174476
mmu05332	Graft-versus-host disease - Mus musculus (house mouse)	46	0.642696118	1.546072001	0.00261561	0.00261561	0.015893898
mmu05146	Amoebiasis - Mus musculus (house mouse)	92	0.56809201	1.427593911	0.003021148	0.003021148	0.017950213
mmu05133	Pertussis - Mus musculus (house mouse)	73	0.584704299	1.453510198	0.003251702	0.003251702	0.018900053
mmu04668	TNF signaling pathway - Mus musculus (house mouse)	113	0.548599877	1.387638879	0.003513703	0.003513703	0.019988369
mmu04390	Hippo signaling pathway - Mus musculus (house mouse)	144	0.526925082	1.341641169	0.003804946	0.003804946	0.021194219
mmu04972	Pancreatic secretion - Mus musculus (house mouse)	90	0.560612845	1.407060729	0.003933831	0.003933831	0.021464942
mmu04115	p53 signaling pathway - Mus musculus (house mouse)	72	0.583091037	1.448487671	0.004067107	0.004067107	0.021748321
mmu05321	Inflammatory bowel disease - Mus musculus (house mouse)	59	0.598295705	1.467063823	0.004620598	0.004620598	0.024221445
mmu04350	TGF-beta signaling pathway - Mus musculus (house mouse)	97	0.551911064	1.389934045	0.00472837	0.00472837	0.024221445
mmu05416	Viral myocarditis - Mus musculus (house mouse)	71	0.578775277	1.436521952	0.004884502	0.004884502	0.024221445
mmu03264	Virion - Flavivirus - Mus musculus (house mouse)	8	-0.836288112	-1.883285573	0.004891969	0.004891969	0.024221445
mmu04510	Focal adhesion - Mus musculus (house mouse)	194	0.506010264	1.29594405	0.00510153	0.00510153	0.024799784
mmu04961	Endocrine and other factor-regulated calcium reabsorption - Mus musculus (house mouse)	54	0.602184098	1.468244717	0.005568158	0.005568158	0.026584816
mmu05143	African trypanosomiasis - Mus musculus (house mouse)	30	0.664298106	1.529108934	0.006091591	0.006091591	0.02857367
mmu04964	Proximal tubule bicarbonate reclamation - Mus musculus (house mouse)	19	0.714896059	1.543991979	0.008058945	0.008058945	0.03715013
mmu04726	Serotonergic synapse - Mus musculus (house mouse)	96	0.541058915	1.362070274	0.00825448	0.00825448	0.037406562
mmu05033	Nicotine addiction - Mus musculus (house mouse)	34	0.639041675	1.492505337	0.009327758	0.009327758	0.041565799

Supplementary Table 2: GSEA of d8 infection vs NI

ID	Description	setSize	enrichmentScore	NES	pvalue	p.adjust	qvalue
mmu05203	Viral carcinogenesis - Mus musculus (house mouse)	178	0.513665392	1.383636254	0.003839	0.003839	0.842478
mmu04810	Regulation of actin cytoskeleton - Mus musculus (house mouse)	208	0.493524828	1.335632523	0.006347	0.006347	0.842478
mmu04014	Ras signaling pathway - Mus musculus (house mouse)	209	0.48336418	1.308357215	0.012593	0.012593	0.842478
mmu05163	Human cytomegalovirus infection - Mus musculus (house mouse)	234	0.470248923	1.276954958	0.018667	0.018667	0.842478
mmu04360	Axon guidance - Mus musculus (house mouse)	169	0.484980175	1.303977964	0.021548	0.021548	0.842478
mmu04721	Synaptic vesicle cycle - Mus musculus (house mouse)	67	0.556504909	1.407079652	0.021673	0.021673	0.842478
mmu05410	Hypertrophic cardiomyopathy - Mus musculus (house mouse)	83	-0.370210484	-1.330305246	0.025	0.025	0.842478
mmu00061	Fatty acid biosynthesis - Mus musculus (house mouse)	16	0.709374143	1.470275485	0.027048	0.027048	0.842478
mmu04979	Cholesterol metabolism - Mus musculus (house mouse)	44	0.587759706	1.419065044	0.028379	0.028379	0.842478
mmu00531	Glycosaminoglycan degradation - Mus musculus (house mouse)	16	0.700747633	1.452395856	0.033745	0.033745	0.842478
mmu04650	Natural killer cell mediated cytotoxicity - Mus musculus (house mouse)	113	0.501837157	1.321980698	0.033795	0.033795	0.842478
mmu05132	Salmonella infection - Mus musculus (house mouse)	243	0.4573578	1.243003105	0.034012	0.034012	0.842478
mmu05231	Choline metabolism in cancer - Mus musculus (house mouse)	96	0.510311172	1.330024295	0.038643	0.038643	0.842478
mmu04072	Phospholipase D signaling pathway - Mus musculus (house mouse)	139	0.483533139	1.288864594	0.041654	0.041654	0.842478
mmu04974	Protein digestion and absorption - Mus musculus (house mouse)	87	-0.345110917	-1.258038798	0.042125	0.042125	0.842478
mmu03013	Nucleocytoplasmic transport - Mus musculus (house mouse)	103	0.499156592	1.306684938	0.045208	0.045208	0.842478
mmu05206	MicroRNAs in cancer - Mus musculus (house mouse)	191	0.460600267	1.243635866	0.047984	0.047984	0.842478
mmu00900	Terpenoid backbone biosynthesis - Mus musculus (house mouse)	23	0.640372802	1.41094219	0.048262	0.048262	0.842478

Supplementary Table 3: GSEA of d30 infection vs NI

ID	Description	setSize	enrichmentScore	NES	pvalue	p.adjust	qvalue
mmu00750	Vitamin B6 metabolism - Mus musculus (house mouse)	9	0.889763083	1.597472342	0.002055639	0.002055639	0.719473756
mmu04972	Pancreatic secretion - Mus musculus (house mouse)	90	0.57867667	1.403104683	0.007276827	0.007276827	0.999003672
mmu03015	mRNA surveillance pathway - Mus musculus (house mouse)	92	0.567289059	1.376906155	0.010954136	0.010954136	0.999003672
mmu00350	Tyrosine metabolism - Mus musculus (house mouse)	24	0.683132706	1.45213159	0.024367275	0.024367275	0.999003672
mmu00534	Glycosaminoglycan biosynthesis - heparan sulfate / heparin - Mus musculus (house mouse)	20	0.704033041	1.455154678	0.025500911	0.025500911	0.999003672
mmu05134	Legionellosis - Mus musculus (house mouse)	60	0.583099035	1.375992004	0.025841156	0.025841156	0.999003672
mmu00591	Linoleic acid metabolism - Mus musculus (house mouse)	24	0.675736247	1.436408975	0.030488523	0.030488523	0.999003672
mmu00430	Taurine and hypotaurine metabolism - Mus musculus (house mouse)	12	0.761691205	1.446521859	0.032017773	0.032017773	0.999003672
mmu04151	PI3K-Akt signaling pathway - Mus musculus (house mouse)	329	0.466708875	1.171914452	0.042	0.042	0.999003672
mmu05014	Amyotrophic lateral sclerosis - Mus musculus (house mouse)	324	0.466917328	1.172104655	0.04390439	0.04390439	0.999003672

Supplementary Table 4: Purity of pDCs

Sample	Purity (%)
HC1	98.9
HC2	96
HC3	99
HC4	91.8
HC5	94.5
HC6	92
HC7	96.9
HC8	99
HC9	97.6
HC10	99
HC11	98.6
HC12	94.2
HC13	96.1
HC14	96.5
HIV1	96.8
HIV2	94.5
HIV3	96.1
HIV4	95.5
HIV5	90.4

Supplementary Table 5: gRNA Sequences

Name	Sequence	PAM
gRNA1	GGGTGTTGGACAAGTTGGTA	TGG
gRNA2	CTTCCAATCACGCGGTGTTT	GGG
gRNA3	GGACTGTACTTGACGATCTG	AGG

Supplementary Table 6: qPCR Primers

Species	Target	Forward Primer	Reverse Primer
Mouse	*Ifna*	TATGTCCTCACAGCCAGCAG	TTCTGCAATGACCTCCATCA
Mouse	*Ldhb*	TTCCTCCAGACTCCGAAAATTG	GTTTCCAGGTGACGTAAGTCAG
Mouse	*Gapdh*	CATGGCCTTCCGTGTTCCTA	CCTGCTTCACCACCTTCTTGAT
Mouse	*Tnfa*	CCCTCACACTCAGATCATCTTCT	GCTACGACGTGGGCTACAG
Human	*GAPDH*	TGATGACATCAAGAAGGTGGTGAAG	TCCTTGGAGGCCATGTGGGCCAT
Human	*LDHB*	TGGTATGGCGTGTGCTATCAG	TTGGCGGTCACAGAATAATCTTT

Supplementary Table 7: Antibodies

Species	Target	Clone	Fluor	Company	Catalogue #	Dilution
Mouse	CD90.2 (Thy-1.2)	30-H12	PerCP-eFluor 710	eBioscience	46-0903-82	1:400
Mouse	CD19	1D3	PerCP/Cy5.5	eBioscience	45-0193-82	1:400
Mouse	NK1.1	PK136	PerCP/Cy5.5	eBioscience	45-5941-82	1:400
Mouse	CD90.2 (Thy1.2)	30-H12	Alexa Fluor 700	BioLegend	105320	1:400
Mouse	CD19	eBio1D3	Alexa Fluor 700	eBioscience	56-0193-82	1:150
Mouse	NK1.1	PK136	Alexa Fluor 700	eBioscience	56-5941-80	1:150
Mouse	Ly-6G/Ly-6C (Gr-1)	RB6-8C5	PerCP/Cy5.5	BioLegend	108428	1:800
Mouse	CD11c	N418	APC	eBioscience	17-0114-81	1:00
Mouse	CD11b	M1/70	PerCP/Cy5.5	eBioscience	45-0112-82	1:200
Mouse	CD11b	M1/70	PE	eBioscience	12-0112-83	1:200
Mouse	CD45R (B220)	RA3-6B2	APC-Cy7	BioLegend	103224	1:200
Mouse	CD317 (BST2, PDCA-1)	eBio129c (129c)	PE	eBioscience	12-3171-82	1:300
Mouse	CD317 (BST2, PDCA-1)	eBio927	PE-Cy7	eBioscience	25-3172-82	1:300
Mouse	CD317 (BST2, PDCA-1)	eBio927	FITC	eBioscience	11-3172-82	1:300
Mouse	CD8a	53-6.7	BUV395	BD Biosciences	563786	1:100
Mouse	CD8a	53-6.7	PerCP/Cy5.5	eBioscience	45-0081-82	1:100
Mouse	CD45.1	A20	PE-CF594	BD Biosciences	562452	1:50
Mouse	CD45.1	A20	PE-Cy7	eBioscience	25-0453-82	1:50
Mouse	CD45.2	104	eFluor 450	eBioscience	48-0454-82	1:50
Mouse	CD45.2	104	APC-eFluor 780	eBioscience	47-0454-82	1:50
Mouse	MHC-II (I-A/I-E)	M5/114.15.2	PerCP-eFluor 710	eBioscience	46-5321-82	1:1000
Mouse	I-A/I-E (MHC-II)	M5/114.15.2	BV650	BioLegend	107641	1:1000
Mouse	CD86	GL-1	BV605	BioLegend	105037	1:100
Mouse	TER-119	TER-119	PerCP/Cy5.5	eBioscience	45-5921-82	1:1000
Mouse	CD127	A7R34	PerCP/Cy5.5	eBioscience	45-1271-82	1:100
Mouse	CD3e	145-2C11	PerCP/Cy5.5	eBioscience	45-0031-82	1:100
Mouse	CD3e	145-2C11	eFluor 450	eBioscience	48-0031-82	1:100
Mouse	CD4	RM4-5	PerCP/Cy5.5	eBioscience	45-0042-82	1:200
Mouse	CD4	RM4-5	BUV737	BD Biosciences	612843	1:200
Human	CD3	UCTH1	Pacific Blue	BioLegend	300418	1:200
Human	CD14	HCD14	Pacific Blue	BioLegend	325615	1:200
Human	CD16	3G8	Pacific Blue	BioLegend	302024	1:200
Human	CD19	HIB19	Pacific Blue	BioLegend	302223	1:200
Human	CD56	MEM-188	Pacific Blue	BioLegend	304629	1:200
Human	HLA-DR	L243	APC-Cy7	BioLegend	307617	1:30
Human	CD11c	B-ly6	BV605	BD Biosciences	563403	1:40
Human	CD123	6H6	PE	BioLegend	306006	1:40
Human	CD304	12C2	BV605	BioLegend	354531	1:40
Human	LDHB	EP1566Y	APC	Abcam	ab310866	1:100
Human	TNFa	Mab11	PerCP/Cy5.5	BioLegend	502926	1:30
Human	IFNa	LT27:295	FITC	Miltenyi Biotec	130-128-082	1:10
