# Supplementary material for: Metabolic deficiencies underlie reduced plasmacytoid dendritic cell IFN-I production following viral infection
Source: Nat Commun. 2025 Feb 7;16:1460. doi: 10.1038/s41467-025-56603-5 (PMC11805920; doi:10.1038/s41467-025-56603-5)
Supplement: Supplementary file 2 — Description of Additional Supplementary Files [file 41467_2025_56603_MOESM2_ESM.pdf]

Description of Additional Supplementary Files

File Name: Supplementary Data 1

Description: Differentially expressed genes for comparison of pDCs from Uninfected Mice or Mice at 24hr post infection with fold change and adjusted p values.

File Name: Supplementary Data 2

Description: Differentially expressed genes for comparison of pDCs from Uninfected Mice or Mice at day 8 post infection with fold change and adjusted p values.

File Name: Supplementary Data 3

Description: Differentially expressed genes for comparison of pDCs from Uninfected Mice or Mice at day 30 post infection with fold change and adjusted p values.

File Name: Supplementary Data 4

Description: Differentially expressed genes associated with their given clusters for associated supplementary Figure 2b.

File Name: Supplementary Data 5

Description: Gene ontology analysis for DEG that are shared by pDC from Day 8 and Day 30 post infection.

File Name: Supplementary Data 6

Description: Differentially expressed genes as determined by microarray for TLR7 knockout and WT compartments of mixed bone marrow chimeras.
