# Supplementary material for: Metabolic deficiencies underlie reduced plasmacytoid dendritic cell IFN-I production following viral infection
Source: Nat Commun. 2025 Feb 7;16:1460. doi: 10.1038/s41467-025-56603-5 (PMC11805920; doi:10.1038/s41467-025-56603-5)
Supplement: Supplementary file 9 — Reporting Summary [file 41467_2025_56603_MOESM9_ESM.pdf]

Corresponding author(s): Elina I. Zuniga

Last updated by author(s): 1/21/2025

Reporting Summary

Nature Portfolio wishes to improve the reproducibility of the work that we publish. This form provides structure for consistency and transparency in reporting. For further information on Nature Portfolio policies, see our Editorial Policies and the Editorial Policy Checklist.

Statistics

For all statistical analyses, confirm that the following items are present in the figure legend, table legend, main text, or Methods section.

n/a	Confirmed	
☐	☒	The exact sample size (*n*) for each experimental group/condition, given as a discrete number and unit of measurement
☐	☒	A statement on whether measurements were taken from distinct samples or whether the same sample was measured repeatedly
☐	☒	The statistical test(s) used AND whether they are one- or two-sided<br>*Only common tests should be described solely by name; describe more complex techniques in the Methods section.*
☐	☒	A description of all covariates tested
☐	☒	A description of any assumptions or corrections, such as tests of normality and adjustment for multiple comparisons
☐	☒	A full description of the statistical parameters including central tendency (e.g. means) or other basic estimates (e.g. regression coefficient) AND variation (e.g. standard deviation) or associated estimates of uncertainty (e.g. confidence intervals)
☐	☒	For null hypothesis testing, the test statistic (e.g. *F*, *t*, *r*) with confidence intervals, effect sizes, degrees of freedom and *P* value noted<br>*Give P values as exact values whenever suitable.*
☒	☐	For Bayesian analysis, information on the choice of priors and Markov chain Monte Carlo settings
☒	☐	For hierarchical and complex designs, identification of the appropriate level for tests and full reporting of outcomes
☒	☐	Estimates of effect sizes (e.g. Cohen's *d*, Pearson's *r*), indicating how they were calculated

Our web collection on statistics for biologists contains articles on many of the points above.

Software and code

Policy information about availability of computer code

Data collection	Data collection for flow cytometry was performed with Everest (Biolegend). Luminescence was performed using SparkControl v3.1. qPCR data collection was performed using Bio-rad CFX-manager 3.1. Seahorse data was collected using XF_Software 1.0, or XF HS Mini Control Software.
Data analysis	Data analysis for flow cytometry was performed with FlowJo v10 (BD Biosciences). Histopathological assessment was performed using QuPath v0.4. All other data analysis was performed in Graphpad Prism 10.

For manuscripts utilizing custom algorithms or software that are central to the research but not yet described in published literature, software must be made available to editors and reviewers. We strongly encourage code deposition in a community repository (e.g. GitHub). See the Nature Portfolio guidelines for submitting code & software for further information.

Data

Policy information about availability of data

All manuscripts must include a data availability statement. This statement should provide the following information, where applicable:

- Accession codes, unique identifiers, or web links for publicly available datasets
- A description of any restrictions on data availability
- For clinical datasets or third party data, please ensure that the statement adheres to our policy

The RNAseq and Microarray data have been deposited in the Gene Expression Omnibus (GEO) database under GSE285244 and GSE285874 at https://www.ncbi.nlm.nih.gov/geo/query/acc.cgi?acc=GSE285244 or https://www.ncbi.nlm.nih.gov/geo/query/acc.cgi?acc=GSE285874 respectively. Other data and reagents are available from the corresponding author on request. All data are included in the Supplementary Information or available from the authors, as are

unique reagents used in this Article. The raw numbers for charts and graphs are available in the Source Data file whenever possible.

Research involving human participants, their data, or biological material

Policy information about studies with human participants or human data. See also policy information about sex, gender (identity/presentation), and sexual orientation and race, ethnicity and racism.

Reporting on sex and gender	ex was not considered in study design, and was determined based on self-reporting. Sex based analyses were not performed as cohort numbers were too small to identify differences based on this factor.
Reporting on race, ethnicity, or other socially relevant groupings	See above.
Population characteristics	*Describe the covariate-relevant population characteristics of the human research participants (e.g. age, genotypic information, past and current diagnosis and treatment categories). If you filled out the behavioural & social sciences study design questions and have nothing to add here, write "See above."*
Recruitment	Blood samples from HIV-negative and HIV-infected volunteers were obtained with consent according to institutional guidelines and the Declaration of Helsinki
Ethics oversight	All human subject studies were approved by the Institutional Review Board (IRB) at Rutgers, the State University of New Jersey, New Jersey Medical School.

Note that full information on the approval of the study protocol must also be provided in the manuscript.

Field-specific reporting

Please select the one below that is the best fit for your research. If you are not sure, read the appropriate sections before making your selection.

☒ Life sciences ☐ Behavioural & social sciences ☐ Ecological, evolutionary & environmental sciences

For a reference copy of the document with all sections, see nature.com/documents/nr-reporting-summary-flat.pdf

Life sciences study design

All studies must disclose on these points even when the disclosure is negative.

Sample size	No statistical methods were used to predetermine sample size. Sample sizes were based on previous experience with the models used.
Data exclusions	No Data were excluded from this study.
Replication	Numbers of replication for each experiment are indicated in the figure legends.
Randomization	Organisms were distributed randomly into treatment or non-treatment groups via random selection of mice from available cages.
Blinding	Investigators were not blinded as this was not possible given the highly technical nature of most experiments. For colon length measurement measurements were performed blinded and this is indicated in the methods.

Reporting for specific materials, systems and methods

We require information from authors about some types of materials, experimental systems and methods used in many studies. Here, indicate whether each material, system or method listed is relevant to your study. If you are not sure if a list item applies to your research, read the appropriate section before selecting a response.

Materials & experimental systems

n/a	Involved in the study	
☐	☒	Antibodies
☐	☒	Eukaryotic cell lines
☒	☐	Palaeontology and archaeology
☐	☒	Animals and other organisms
☒	☐	Clinical data
☒	☐	Dual use research of concern
☒	☐	Plants

Methods

n/a	Involved in the study	
☒	☐	ChIP-seq
☐	☒	Flow cytometry
☒	☐	MRI-based neuroimaging

Antibodies

Antibodies used	Species Target Clone Fluor Company Catalogue # Dilution<br>Mouse CD90.2 (Thy-1.2) 30-H12 PerCP-eFluor 710 eBioscience 46-0903-82 1:400<br>Mouse CD19 1D3 PerCP/Cy5.5 eBioscience 45-0193-82 1:400<br>Mouse NK1.1 PK136 PerCP/Cy5.5 eBioscience 45-5941-82 1:400<br>Mouse CD90.2 (Thy1.2) 30-H12 Alexa Fluor 700 BioLegend 105320 1:400<br>Mouse CD19 eBio1D3 Alexa Fluor 700 eBioscience 56-0193-82 1:150<br>Mouse NK1.1 PK136 Alexa Fluor 700 eBioscience 56-5941-80 1:150<br>Mouse Ly-6G/Ly-6C (Gr-1) RB6-8C5 PerCP/Cy5.5 BioLegend 108428 1:800<br>Mouse CD11c N418 APC eBioscience 17-0114-81 1:00<br>Mouse CD11b M1/70 PerCP/Cy5.5 eBioscience 45-0112-82 1:200<br>Mouse CD11b M1/70 PE eBioscience 12-0112-83 1:200<br>Mouse CD45R (B220) RA3-6B2 APC-Cy7 BioLegend 103224 1:200<br>Mouse CD317 (BST2, PDCA-1) eBio129c (129c) PE eBioscience 12-3171-82 1:300<br>Mouse CD317 (BST2, PDCA-1) eBio927 PE-Cy7 eBioscience 25-3172-82 1:300<br>Mouse CD317 (BST2, PDCA-1) eBio927 FITC eBioscience 11-3172-82 1:300<br>Mouse CD8a 53-6.7 BUV395 BD Biosciences 563786 1:100<br>Mouse CD8a 53-6.7 PerCP/Cy5.5 eBioscience 45-0081-82 1:100<br>Mouse CD45.1 A20 PE-CF594 BD Biosciences 562452 1:50<br>Mouse CD45.1 A20 PE-Cy7 eBioscience 25-0453-82 1:50<br>Mouse CD45.2 104 eFluor 450 eBioscience 48-0454-82 1:50<br>Mouse CD45.2 104 APC-eFluor 780 eBioscience 47-0454-82 1:50<br>Mouse MHC-II (I-A/I-E) M5/114.15.2 PerCP-eFluor 710 eBioscience 46-5321-82 1:1000<br>Mouse I-A/I-E (MHC-II) M5/114.15.2 BV650 BioLegend 107641 1:1000<br>Mouse CD86 GL-1 BV605 BioLegend 105037 1:100<br>Mouse TER-119 TER-119 PerCP/Cy5.5 eBioscience 45-5921-82 1:1000<br>Mouse CD127 A7R34 PerCP/Cy5.5 eBioscience 45-1271-82 1:100<br>Mouse CD3e 145-2C11 PerCP/Cy5.5 eBioscience 45-0031-82 1:100<br>Mouse CD3e 145-2C11 eFluor 450 eBioscience 48-0031-82 1:100<br>Mouse CD4 RM4-5 PerCP/Cy5.5 eBioscience 45-0042-82 1:200<br>Mouse CD4 RM4-5 BUV737 BD Biosciences 612843 1:200<br>Human CD3 UCTH1 Pacific Blue BioLegend 300418 1:200<br>Human CD14 HCD14 Pacific Blue BioLegend 325615 1:200<br>Human CD16 3G8 Pacific Blue BioLegend 302024 1:200<br>Human CD19 HIB19 Pacific Blue BioLegend 302223 1:200<br>Human CD56 MEM-188 Pacific Blue BioLegend 304629 1:200<br>Human HLA-DR L243 APC-Cy7 BioLegend 307617 1:30<br>Human CD11c B-ly6 BV605 BD Biosciences 563403 1:40<br>Human CD123 6H6 PE BioLegend 306006 1:40<br>Human CD304 12C2 BV605 BioLegend 354531 1:40<br>Human LDHB EP1566Y APC Abcam ab310866 1:100<br>Human TNFa Mab11 PerCP/Cy5.5 BioLegend 502926 1:30<br>Human IFNa LT27:295 FITC Miltenyi Biotec 130-128-082 1:10
Validation	Antibodies used were validated by the manufacturer.

Eukaryotic cell lines

Policy information about cell lines and Sex and Gender in Research

Cell line source(s)	BHK, Vero, 293T were originally obtained from the ATCC and maintained in the Zuniga Lab. L929 cells were provided by Juan Carlos de la Torre.
Authentication	Cell lines used were not authenticated.
Mycoplasma contamination	All cell lines used were tested for mycoplasma contamination and were negative.
Commonly misidentified lines (See ICLAC register)	No commonly misidentified cell lines were used in this study.

Animals and other research organisms

Policy information about studies involving animals; ARRIVE guidelines recommended for reporting animal research, and Sex and Gender in Research

Laboratory animals	Six to eight-week-old female C57BL/6 (Strain #000664), B6 CD45.1 (Strain #002014), sham-operated and ADX mice were purchased

from The Jackson Laboratory. TLR7-/- mice (Strain #008380) and BDCA2-DTR mice (Strain #014176) were purchased from The Jackson laboratory and bred in house. LDHB-/- mice (C57BL/6NTac-Ldhbtm1a(KOMP)Wtsi) were generated by the international mouse phenotyping consortium (IMPC, www.mousephenotype.org) and procured from the Knockout Mouse Consortium (KOMP). All mice were housed under specific-pathogen-free conditions at the University of California, San Diego at an ambient temperature of 22-25C and ambient humidity with a 12 hour day-night cycle. For all experiments experimental and control animals were cohoused up until the point of infection, at which point infected and uninfected groups were kept in separate cages. When two infected groups were compared, they were housed separately through the course of infection. For pDC depletion mixed BM chimeras of 50% BDCA2-DTR and 50% either WT or LDHB-/- BM were treated with diphtheria toxin (DT) (Sigma-Aldrich, Catalogue #:D0564)70 Mice were given 200 ng of DT by intraperitoneal injection daily starting from two days prior to MHV infection, and up to endpoint (48 hrs p.i.). Mice were euthanized via CO2 chamber per the recommendations of the UCSD Institutional Animal Care and Use Committee (IACUC). Mouse handling and experiments conformed to the requirements of the National Institute of Health and the IACUC Guidelines of UC San Diego. Permission was granted to perform experiments as described by the UC San Diego IACUC. Unless stated otherwise, experiments were initiated in mice (female and male) at 7-12 weeks of age.

Wild animals: No wild animals were used in this study.

Reporting on sex: Sex was not considered in study design. Experiments use animals of both sexes.

Field-collected samples: This study did not involve samples collected in the field.

Ethics oversight: Mouse handling and experiments conformed to the requirements of the National Institute of Health and the IACUC Guidelines of UC San Diego. Permission was granted to perform experiments as described by the UC San Diego IACUC.

Note that full information on the approval of the study protocol must also be provided in the manuscript.

Plants

Seed stocks: No plants were used in this study.

Novel plant genotypes: No plants were used in this study.

Authentication: No plants were used in this study.

Flow Cytometry

Plots

Confirm that:

- [x] The axis labels state the marker and fluorochrome used (e.g. CD4-FITC).
- [x] The axis scales are clearly visible. Include numbers along axes only for bottom left plot of group (a 'group' is an analysis of identical markers).
- [x] All plots are contour plots with outliers or pseudocolor plots.
- [x] A numerical value for number of cells or percentage (with statistics) is provided.

Methodology

Sample preparation: Spleen samples were disaggregated using Collagenase D, then filtered, treated with ACK to remove red blood cells, and filtered again. For sorting or prior to stimulation pDC were enriched by removing T, and B cells using an EasySep RapidSpheres Isolation Kit (Stemcell Technologies) with biotin conjugated anti-Thy1.2 (53-2.1) and anti-CD19 (6D5).

Instrument: Samples for analysis were collected on a ZE5 (Biorad). Samples sorted were collected on an Aria Fusion II (BD Biosciences).

Software: All analysis was performed using FlowJo Software (BD Biosciences).

Cell population abundance: Purity and post sort viability was determined using a distinct flow cytometer from that used in sorting. Purity was evaluated for pDC, cDC1, or cDC2 as % of cells in the same gate as the given cell population from an unsorted sample.

Gating strategy: In spleen pDC were gated as Lineage (CD19, Thy1.2, NK1.1) negative, CD11c+, B220+, BST2+ cells, cDC1 were gated as Lineage negative, CD11c+, B220-, CD11b-,CD8a+, cDC2 were gated as Lineage negative, CD11c+, B220-, CD11b+, CD8a-. In Flt3L culture pDC were gated as CD11c+, CD11b-, B220+, BST2+ and cDC2 were gated as CD11c+, CD11b+, B220-. Where applicable congenic markers (CD45.1, CD45.2) were analyzed after gating for cell type.

- [x] Tick this box to confirm that a figure exemplifying the gating strategy is provided in the Supplementary Information.
